# A matter of new life and cell death: programmed cell death in the mammalian ovary

**DOI:** 10.1186/s12929-024-01017-6

**Published:** 2024-03-20

**Authors:** Mikhail S. Chesnokov, Aygun R. Mamedova, Boris Zhivotovsky, Gelina S. Kopeina

**Affiliations:** 1 Faculty of Medicine, MV Lomonosov Moscow State University, Moscow, Russia; 2 Engelhardt Institute of Molecular Biology, Russian Academy of Sciences, Moscow, Russia; 3 Institute of Environmental Medicine, Karolinska Institute, Stockholm, Sweden; 4 Present Address: Centro Nacional de Investigaciones Oncológicas, Madrid, Spain

**Keywords:** Ovarian development, Programmed cell death, Primordial germ cells, Follicular atresia, Luteolysis, Polycystic ovary syndrome, Premature ovarian insufficiency, Ovarian tumors

## Abstract

**Background:**

The mammalian ovary is a unique organ that displays a distinctive feature of cyclic changes throughout the entire reproductive period. The estrous/menstrual cycles are associated with drastic functional and morphological rearrangements of ovarian tissue, including follicular development and degeneration, and the formation and subsequent atrophy of the corpus luteum. The flawless execution of these reiterative processes is impossible without the involvement of programmed cell death (PCD).

**Main text:**

PCD is crucial for efficient and careful clearance of excessive, depleted, or obsolete ovarian structures for ovarian cycling. Moreover, PCD facilitates selection of high-quality oocytes and formation of the ovarian reserve during embryonic and juvenile development. Disruption of PCD regulation can heavily impact the ovarian functions and is associated with various pathologies, from a moderate decrease in fertility to severe hormonal disturbance, complete loss of reproductive function, and tumorigenesis. This comprehensive review aims to provide updated information on the role of PCD in various processes occurring in normal and pathologic ovaries. Three major events of PCD in the ovary—progenitor germ cell depletion, follicular atresia, and corpus luteum degradation—are described, alongside the detailed information on molecular regulation of these processes, highlighting the contribution of apoptosis, autophagy, necroptosis, and ferroptosis. Ultimately, the current knowledge of PCD aberrations associated with pathologies, such as polycystic ovarian syndrome, premature ovarian insufficiency, and tumors of ovarian origin, is outlined.

**Conclusion:**

PCD is an essential element in ovarian development, functions and pathologies. A thorough understanding of molecular mechanisms regulating PCD events is required for future advances in the diagnosis and management of various disorders of the ovary and the female reproductive system in general.

## Background

Regulated cell death (RCD) is an essential part оf the developmental process. The controlled elimination of unwanted cells occurs in virtually any tissue of the body, from skin to the nervous system [1]. In general, RCD might occur on three occasions: as a response to irreparable cell damage (mutations, DNA damage, metabolic impairments, viral infections, etc.), as a mechanism of tissue homeostasis (e.g., to counterbalance constant proliferation and renewal of epithelial tissues), or as an innate process of organ development and functioning (removal of superfluous cells or cells that have accomplished their task) [2]. The second and third cases are together called programmed cell death (PCD) and involve very precise and intricate regulatory mechanisms because the impairments of well-organized cell clearance in normal tissues could easily result in long list of pathologies.

In addition to well-known processes during embryogenesis, such as the development of the central nervous system [3] and finger formation [4], PCD plays a very important role during juvenile and adult life. One of the most important cases of post-embryonic PCD takes place in the ovary and is directly connected to reproductive function, pregnancy, and the estrous/menstrual cycles. The cyclic nature of reproductive processes and their absolute importance for the survival of the species require an intricate regulatory mechanism(s) that should be able to sustain the continuous cycles of the “cell proliferation-differentiation-death-recovery” process.

This review aims to provide current information on the main aspects of PCD involvement in the functions and biological processes in the normal ovary and to highlight PCD abnormalities associated with various ovarian pathologies. We performed an extensive analysis of the published information, paying special attention to the detailed description of molecular mechanisms regulating PCD and the survival of ovarian cells. The established major role of apoptosis is discussed, while also pinpointing the recent discoveries in non-apoptotic modes of PCD (autophagy, necroptosis and ferroptosis).

## The mammalian ovary: from structure to functions

**Fig. 1:**
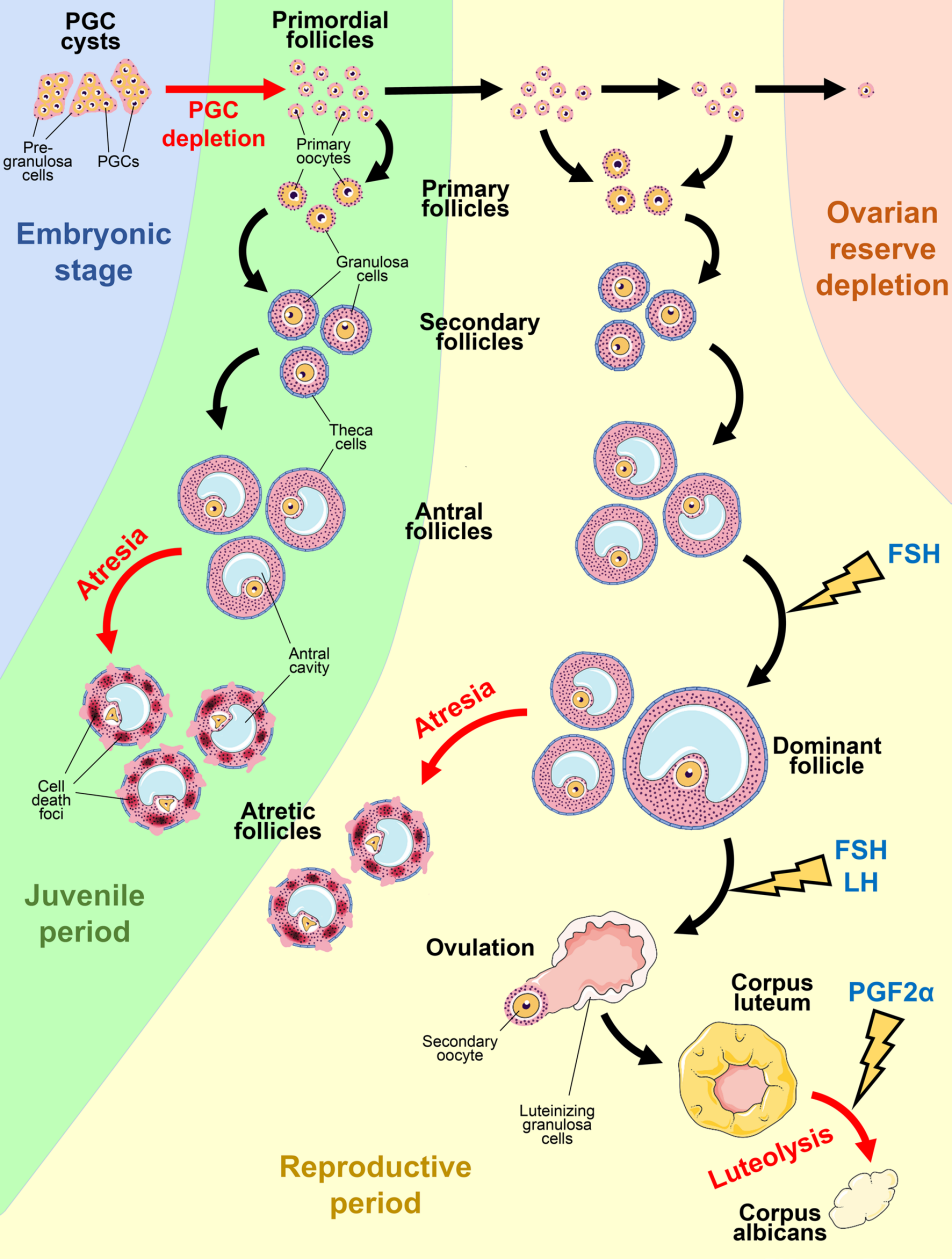
The essential steps of development and degeneration of follicles and the CL in a mammalian ovary. PGC cysts form in the embryonic period (blue area). After the PGC depletion before birth, the primordial follicles start to gradually develop into antral follicles. During the juvenile period (green area) all antral follicles undergo atresia. During the reproductive period (yellow area) a surge of FSH rescues the dominant follicle, while smaller antral follicles degenerate via atresia. Dominant follicles next undergo ovulation stimulated by FSH and LH and transform into CL , which subsequently involutes into corpus albicans in a PGF2α-dependent manner. Primordial follicles are repeatedly recruited until the whole ovarian reserve is depleted (pink area) and the reproductive period ends. The black arrows indicate survival and development, the red arrows indicate degradation events, and the lightning symbols indicate the crucial involvement of endocrine regulators

The first discernible germline cells in the developing embryo are called “primordial germ cells” (PGCs). During the early stages of embryogenesis, these cells migrate from the endoderm to the mesonephros, where epithelial, mesenchymal, and blood vessel cells start to form the body of the fetal ovary. PGCs actively proliferate during the migration and for some time afterward, forming multicellular clusters called “PGC cysts” (Fig. 1) [5, 6]. The cells in these cysts are connected with each other through cytoplasmic intercellular bridges [6]. At the same time, they interact with a specific subpopulation of epithelial cells called pre-granulosa cells to form the initial complexes that will later develop into ovarian follicles [5]. The proliferation of PGCs ultimately concludes in a meiotic division that generates the primary oocytes; however, meiosis is halted at prophase I due to control from the surrounding pre-granulosa cells [7, 8]. The primary oocytes reside in this state called “the dictyate” throughout the rest of embryonic development and a major part of adult life, only resuming their meiotic division in response to stimulation exerted by other elements of the reproductive system.

The first massive wave of PCD is observed in the developing ovary [9]. Up to 80% of PGCs and primary oocytes are eliminated, while only a minor part of the initial population survives [5]. Pre-granulosa cells associated with dying PGCs survive and are relocated to other, viable PGCs and primary oocytes, resulting in a drastic increase in the ratio of pre-granulosa cells to PGCs/oocytes [10]. As a result, each primary oocyte becomes surrounded by a single layer of flattened pre-granulosa cells and a thin layer of basal membrane, forming a structure known as “primordial follicle” [11]. These follicles remain dormant until birth and through the juvenile period until specific stimulation that promotes their further development [12]. In rodents, the process of primordial follicle formation and PGC depletion is extended through the first week after birth [13].

At the moment of birth, the ovary consists of three distinct compartments: the superficial epithelium (one layer of cubic epithelial cells), the cortex (a major part of the ovary that contains primordial and developing follicles), and the medulla (mesenchymal stromal tissue that houses numerous blood vessels) [14]. It had been assumed that the oocytes present at birth represent the entire reserve of potential ova available for the duration of life. However, this dogma was disproved by demonstrating that germ cells can enter meiosis and produce new oocytes and follicles during the reproductive age [15, 16].

After the formation of primordial follicles, a process termed “initial recruitment” starts and continues throughout the entire reproductive age [17]. Primordial follicles are stimulated by the local environment and transform into “primary follicles”, which are recognized by an increase in the oocyte size and the proliferation of the surrounding layer of granulosa cells. The oocyte is separated from the granulosa cells by the zona pellucida, which is filled with glycoprotein matrix, and there are long cytoplasmic protrusions connecting granulosa cells to the oocyte [18–20]. The ongoing growth results in development of “secondary follicle” that exhibits an even larger oocyte and two or more layers of granulosa cells surrounded by theca cells [19]. Around this point, the mature granulosa cells start to express the receptors for follicle-stimulating hormone (FSH), making primary and secondary follicles sensitive to pituitary stimuli [21]. The formation of a fluid-filled cavity marks the antral stage; the antral follicles are ready for terminal maturation—if they can survive [19].

The second crucial case of PCD occurs at this stage and is called “atresia”, a widespread degeneration of antral follicles. Follicular atresia starts with several dying granulosa cells and rapidly spreads across the entire follicle, efficiently obliterating it within several days. Atresia is a default outcome of follicle development, and most follicles degenerate without reaching maturation [17, 19]. The main factor that rescues the follicle is a surge of FSH excreted by the pituitary gland during each estrous/menstrual cycle after the onset of puberty. These repeated events are called “cyclic recruitment” and result in the selection of several dominant antral follicles based on their sensitivity to FSH and other hormones (Fig. 1). The number of dominant follicles is specific for each species and is associated with the size of the offspring (e.g., there is usually only one dominant follicle in a human ovary) [17, 19, 22].

The dominant follicle reaches the terminal maturation state, when granulosa cells start to produce high levels of estradiol; the follicle also becomes extremely responsive to FSH and luteinizing hormone (LH), another pituitary gonadotropin [22]. At some point, the inhibitory effect is exerted by the dominant follicle(s) upon the pituitary gland and results in suppression of FSH secretion. The dominant follicle develops further with support from LH and estradiol; less mature follicles are unable to survive the decline in FSH levels and undergo atretic degeneration [22, 23]. LH stimulation of granulosa cells lifts the prophase I arrest, and the first meiotic division occurs, resulting in formation of a “secondary oocyte” [24]. The final step of the follicle development is associated with a simultaneous surge in FSH, LH, and estradiol levels that promotes follicle rupture and release of the oocyte [22, 25]. The secondary oocyte subsequently interacts with a spermatozoid, undergoes the second meiotic division, develops into a mature ovum, and is fertilized to form a zygote [24].

The granulosa cells of the ruptured follicle do not simply degrade after ovulation; rather, their function is to provide a favorable environment for the embryo to develop and successfully implant into the uterus. This function is achieved through their terminal differentiation into the corpus luteum (CL), a temporary gland inside the ovarian tissue. Luteal cells produce high levels of progesterone that affect the endometrium of the uterus and promote the implantation of the blastocyst and its further development into the embryo [26].

The final fate of luteal cells is determined by the success of embryo implantation into the endometrium. If the implantation fails, then CL cells quickly die in a process called “luteolysis” that starts within several days after ovulation (Fig. 1) [26]. In many species, CL regression is primarily stimulated by prostaglandin F2α (PGF2α) secreted by the uterus. However, luteolysis in primates (including humans) is not dependent on PGF2α produced by the uterus; it is supposedly caused by a decrease in LH levels [27, 28]. If there is successful embryo implantation and pregnancy, then the lifespan of the CL is extended due to the effect of chorionic gonadotropin (CG, primates and equine) or prolactin (rodents) secreted by the implanted blastocyst or decidua, respectively [29]. The developing placenta takes over the synthesis of progesterone, rendering the CL redundant; it then undergoes prompt destruction through PCD [27]. This third major PCD event concludes the long chain of changes initiated during the cyclic recruitment of follicles and “resets” the ovary, preparing it for a new menstrual/estrous cycle.

Thus, PCD plays an essential role in both the development and normal physiology of the ovary, allowing the organism to successfully progress through multiple cycles of reproduction. Below the most common types of PCD are presented with information regarding their involvement in oocyte depletion, follicular atresia, and luteolysis, as well as various ovarian pathologies.

## Common types of PCD observed in the ovary

The understanding of RCD has evolved tremendously from the most basic observations to the extremely sophisticated models describing the internal molecular cross-talk between multiple modes of cell death [2, 30]. For a long time, apoptosis had been believed to be the only type of PCD/RCD, but studies in recent decades have defined multiple other ways cells are eliminated and have brought them into the spotlight. These types of RCD are caused by different stimuli and are executed through different signaling pathways. They also differ in the effects exerted upon their environment, from relatively clean autophagy-dependent death to disruptive and inflammation-inducing pyroptosis [2]. Below is a brief description of the types of RCD that have been reported to occur in the ovarian cells.

### Apoptosis

Apoptosis is the most common and best described mode of RCD that is often considered to be the “default” way for cells to die. Morphologic changes observed in apoptotic cells include chromatin condensation, fragmentation of the nucleus, membrane blebbing, reduction of the cytoplasm volume, and general cell shrinkage. The cell ultimately breaks down into small membrane-encapsulated apoptotic bodies, which undergo phagocytosis by macrophages or neighboring cells [31].

Apoptosis is orchestrated by activation of a family of proteases called caspases. These enzymes are evolutionally conserved and functionally divided into three major groups: apoptosis initiators (caspase-8, caspase-9, and caspase-10), apoptosis executioners (caspase-3, caspase-6, and caspase-7), and inflammatory caspases (caspase-1, caspase-4, caspase-5, and caspase-11) [32]. Caspase-2 stands as a unique family member as its involvement in cell death and other aspects of cell physiology is a matter of great debate [33, 34]. Various caspases facilitate the signal transduction along two major apoptosis pathways: intrinsic and extrinsic.

Intrinsic (“mitochondrial”) apoptosis activates in conditions of nutrient deprivation, growth factor shortage, detachment from the matrix and other cells, cytotoxic stress, and other non-specific factors. The subsequent overexpression and activation of BH3-only proteins belonging to the BCL-2 family (BIM, NOXA, BBC3/PUMA, and BAD) results in neutralization of pro-survival BCL-2-like proteins (BCL-2, BCL-XL, BCL-2A1, and MCL-1) and activation of the BAX/BAK complexes, which permeabilize the outer mitochondrial membrane [35]. Mitochondrial cytochrome *c* is released into the cytoplasm and forms (in presence of dATP) the apoptosome complex with Apoptotic Peptidase Activating Factor 1 (APAF1) and pro-caspase-9. Activated caspase-9 proteolytically cleaves and activates caspases-3 and caspase-7, which in turn cleave hundreds of substrate proteins and promote the terminal stages of apoptosis [32].

Extrinsic apoptosis is actively stimulated through “death receptor” molecules on the cell surface. These receptors belong to the tumor necrosis factor (TNF) superfamily and include CD95/Fas/Apo-1, TNFR1, TRAIL receptors, and other proteins [36]. Ligand binding results in the assembly of a protein complex that includes the receptor itself, the adaptor proteins FADD/TRADD and pro-caspase-8, and triggers caspase-8 processing [32, 36]. Active caspase-8 directly cleaves and activates executioner caspases. Additionally, caspase-8 can cleave BID and thus promote BAX/BAK activation, switching the signal transduction toward the intrinsic pathway [37].

The molecular machinery controlling apoptosis initiation and progression is very complex, and the role of its specific components can vary considerably in different cells and tissues. The development of multiple experimental models that recapitulate the inactivation or hyperactivation of individual genes and proteins in a tissue-specific manner allowed to thoroughly unravel this complicated network regarding ovary-specific processes [38].

### Autophagy

Autophagy is a permanently operating process in the living cell that provides the removal of non-functional or harmful molecules and organelles, their degradation, and recycling. It is, in general, a pro-survival mechanism that sustains cell renewal and stress resistance. However, in certain conditions RCD relies on autophagic proteins and pathways and should be considered true autophagy-dependent cell death (ADCD) [2, 39].

Macroautophagy (usually referred to as “autophagy” in general) is the most common type of autophagy and is facilitated by autophagosomes, which engulf the organelles and aggregates, separating them from the cytoplasm. They later fuse with lysosomes to form autophagolysosomes for disintegration and reabsorption of useful material. Microautophagy involves direct sequestration of cytosolic content by the lysosome, and chaperone-mediated autophagy is associated with selective transfer of protein molecules into the lysosome. The affected cells appear to be filled with vesicles encapsulating fragments of other organelles [39].

Autophagy is promoted in response to a multitude of stimuli such as stress, various forms of starvation, hypoxia, infections, and intracellular disturbances [40]. Different signaling proteins including phosphatidylinositol 3-kinase (PI3K), a serine/threonine-specific protein kinase (AKT), mammalian target of rapamycin (mTORC1) kinase, and protein kinase AMP-activated catalytic subunit alpha-1 (AMPK) converge on the Unc-51-like autophagy activating kinase (ULK) protein complex. Activated ULK phosphorylates BECN1 and other targets that constitute the PI3KC3 complex associated with pre-autophagosomal membrane structures in the endoplasmic reticulum (ER) [41]. The following steps involve a large family of ATG proteins and the microtubule-associated protein 1 light chain (MAP1LC3 or LC3) protein. Pre-autophagosomal membranes bind the ATG5-ATG12-ATG16 complex and LC3 modified with phosphatidylethanolamine (also known as LC3-II) by ATG3, ATG4, and ATG7. These membranes are called “phagophores” and are ready to engulf their substrates and close into an autophagosome. The final step—fusion of an autophagosome and a lysosome—is controlled by LAMP1 and LAMP2 [40]. Lysosome-targeting compounds like bafilomycin A1 and chloroquine prevent autophagolysosome maturation and inhibit autophagy halfway through it, keeping the cellular contents sequestered but not degraded [42].

While autophagic activity is often increased in the cells undergoing RCD, it can be an attempt to attenuate the stressful conditions and rescue the cell from death. On the other hand, autophagy-related proteins interact with many complexes regulating apoptosis (FADD, caspase-8, MCL-1, BCL-XL), necroptosis (RIPK1, RIPK3), and ferroptosis (ferritin), and can actively promote either apoptotic or necrotic cell death [43]. It is very important to define ADCD as a type of cell death that essentially requires activation of autophagic machinery and can be inhibited by autophagy inactivation [2]. Up to date, ADCD in mammalian tissues has only been confirmed in several specific cases, which do not allow yet to build a general scheme of autophagy involvement into RCD or properly define its physiological impact [43]. Nevertheless, the accumulating knowledge of tight links between autophagy and RCD in various tissues and conditions invokes increasing interest in this area. This review aims to collect and organize the current evidence of how autophagy may impact death and survival of ovarian cells, highlighting the events potentially occurring through ADCD.

### Necroptosis

Necrotic cell death is much more chaotic than apoptosis and was considered an uncontrolled way of accidental cell death until two seminal reports described necrosis induction in glutamate-treated neurons [44] and a switch from apoptosis to necrosis-like cell death in murine thymocytes [45]. Since then, several subtypes of regulated necrotic death have been described, with necroptosis being the first of them. Cells undergoing necroptosis display very distinct morphology: a breach of the cellular membrane results in prominent cell swelling without visible chromatin condensation in the nucleus. The content of the dying cell is released into the surrounding space and induces an inflammatory response in the adjacent cells [46].

Necroptosis progression is regulated by a pathway that relies on three key proteins: receptor interacting serine/threonine kinase 1/3 (RIPK1, RIPK3), and mixed lineage kinase domain like pseudokinase (MLKL) [47]. It is most often initiated by ligands of TNF superfamily receptors (TNF1α, FasL, and TRAIL) but can also be induced by bacterial or viral infections and chemical agents [47]. TNF-associated induction is the most well characterized, and its initial steps have significant overlap with the extrinsic apoptosis pathway. Activated TNF receptors assemble Complex I that consists of TRADD, TRAF2, RIPK1, and inhibitor of apoptosis protein (cIAP1/2) proteins and promotes survival through the NFκB pathway [48]. In the absence of cIAPs, Complex I becomes unstable, and RIPK1 binds to FADD and caspase-8 instead, forming Complex IIb that can promote caspase-8-dependent apoptosis [49]. However, if caspase-8 activity (or caspase activity in general) is inhibited, then RIPK1 undergoes autophosphorylation and assembles Complex IIc (“necroptosome”) together with RIPK3 and MLKL. Subsequent RIPK3 and MLKL phosphorylation results in MLKL oligomerization and translocation to the cytoplasmic membrane, where it forms transmembrane channels and facilitates loss of membrane integrity, condemning the cell to necroptotic death [50]. Due to its disruptive effects, necroptosis is not observed very often in normal tissues, but the aberrations of necroptotic molecular machinery can be associated with various pathologies.

### Ferroptosis

Ferroptosis as a separate, iron-dependent mode of cell death was first described in 2012 and integrated earlier observations of non-apoptotic cell death associated with ions of iron, reactive oxygen species (ROS), and metabolic effects [51, 52]. The most distinct morphologic feature of ferroptosis is mitochondria shrinkage combined with cristae reduction and an increase in membrane density [51]. The plasma membrane integrity and nucleus structure are preserved until the very late stages when the dying cells acquire a necrotic morphology [46, 53].

Ferroptosis is characterized by the peroxidation of polyunsaturated fatty acids (PUFAs) incorporated into membrane phospholipids. Some monounsaturated fatty acids (MUFAs) have been shown to exert the opposite, anti-ferroptotic effects. The role of PUFAs in ferroptosis is highly dependent on their location, and the ER is the most crucial place where PUFA peroxidation occurs [52]. Peroxidation of membrane-bound PUFAs can be initiated by iron-dependent lipoxygenases and is promoted by free Fe^2+^ through the Fenton reaction, which produces hydroxyl radicals. These processes define the importance of iron turnover during ferroptosis initiation. Intracellular iron is deposited in complexes with ferritin or glutathione (GSH), and autophagy-dependent ferritin degradation is an important step during ferroptosis progression [52, 54]. GSH depletion also increases Fe^2+^ availability, but its major role in ferroptosis regulation is carried out through glutathione peroxidase 4 (GPX4), a GSH-dependent peroxidase that protects membrane lipids from oxidation by ROS [55]. GSH is synthesized from cystine imported into the cell via the x_c_^−^ system, and the first described ferroptosis inductors, erastin and RAS-selective lethal (RSL3) compound, are inhibitors of the x_c_^−^ system and GPX4, respectively [56, 57]. Overall, ferroptosis is a relatively new type of RCD which is being actively explored.

## Regulation of PCD during embryonic and juvenile development

**Fig. 2:**
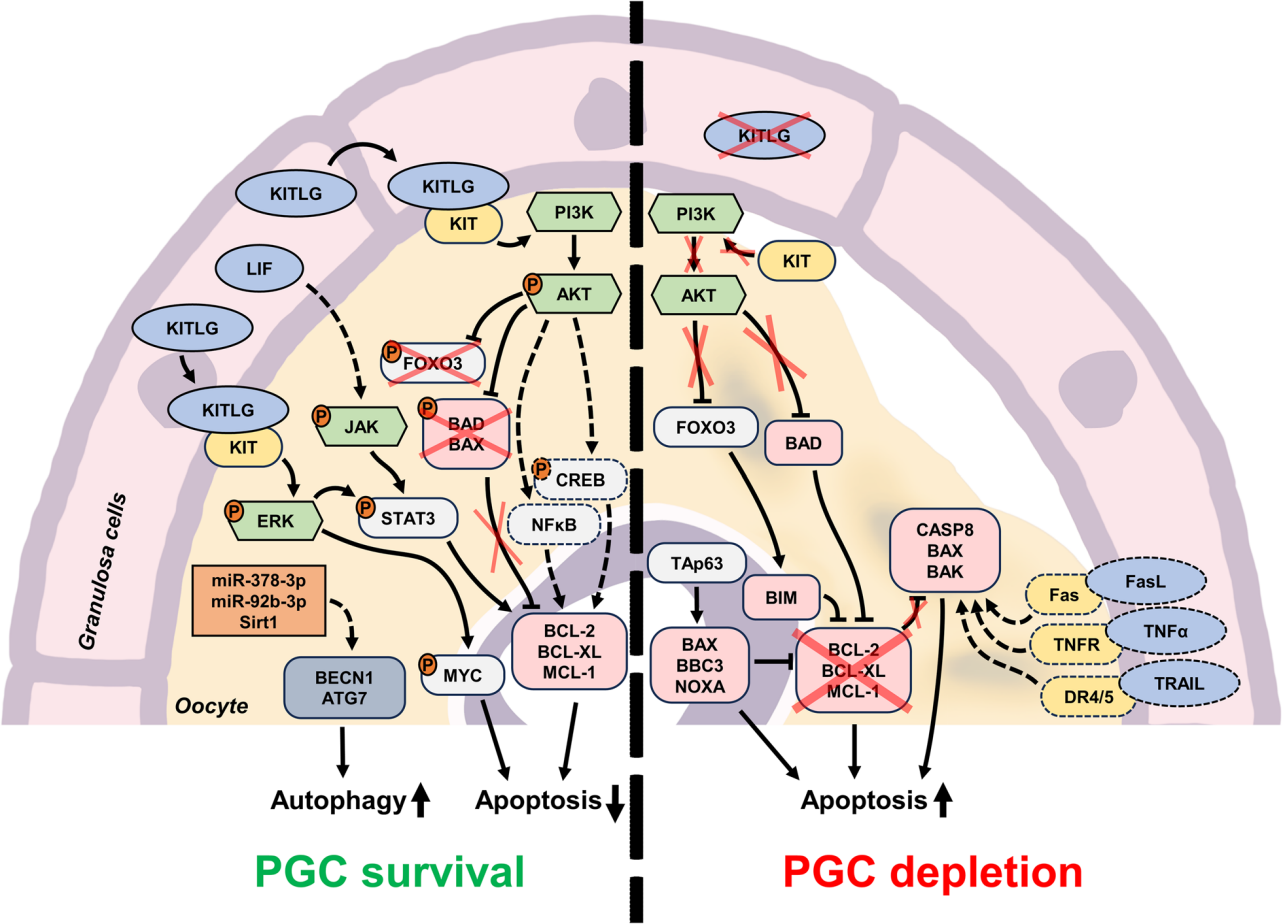
Major molecular mechanisms and regulatory elements involved in the control of PGC depletion process in the embryonic ovary. The pathways regulated by KITLG and LIF, which are produced by granulosa cells, promote PGC survival through inhibition of apoptosis and stimulation of autophagy (left side of the figure). In the absence of KITLG, the intrinsic apoptosis pathway (with potential involvement of extrinsic apoptosis regulators) induces PGC death through apoptosis (right side of the figure). Orange circles with the letter “P” indicate protein phosphorylation, red crosses indicate the lack or suppression of certain regulators, and dashed elements indicate the regulators and connections that are not definitively confirmed up to date

The loss of PGCs during the formation of primordial follicles marks the first significant PCD event in the developing ovary [5, 9]. It is observed in all studied mammals, including mice, rats, and humans [7, 58, 59]. In human ovaries, the total number of primary oocytes decreases from around 7 million cells in a 5-month-old embryo to 1–2 million cells at birth and 300,000 cells at the age of 7 years [59]. Three hypotheses explaining PGC depletion have been proposed and supported by experimental data: (I) degeneration of excess PGCs that did not attract enough pre-granulosa cells and growth factors (“Apoptosis” section [60–62]), (II) lethal DNA damage during crossover step in the meiosis (“Apoptosis” section [63–65]), and (III) sacrifice of normal PGCs to allow the surviving ones to feed on their cytoplasmic contents (“Autophagy” section [6, 66, 67]). The current knowledge of PCD regulation in the developing ovary is discussed below, and the most important regulatory pathways are summarized in Fig. 2.

### Apoptosis

The vast majority of PGCs and primary oocytes undergo caspase-dependent apoptosis [68, 69]. The survival and proliferation of PGCs are crucially dependent on external signaling molecules such as KIT ligand (KITLG (SCF)), Leukemia inhibitory factor (LIF), Insulin like growth factor 1 (IGF1), retinoic acid, and cAMP activators [70, 71]. The presence of KITLG and LIF is essential to prevent PGC apoptosis in vitro [60], and both factors are secreted by pre-granulosa and granulosa cells, promoting PGC survival in vivo [61, 62]. The KIT receptor (also known as c-Kit and CD117) on the surface of PGCs acts through the PI3K/AKT and MEK/ERK signaling pathways [72], and its knockout results in complete oocyte degradation by the moment of birth [73]. The exact pathways facilitating LIF signaling in PGCs have not yet been determined; however, LIF can activate the Janus kinase/Signal transducer and activator of transcription 3 (JAK/STAT3) pathway in porcine oocytes [74] and human granulosa cells [75]. Evidence from other models suggests that STAT3 activation should confer an anti-apoptotic effect [76]. Both the PI3K/AKT and STAT3 pathways may promote the expression of BCL-2, BCL-XL, and MCL-1 [76, 77]. Moreover, AKT can phosphorylate and inactivate the proapoptotic BAX and BAD proteins [77, 78] and the Forkhead box O3 (FOXO3) transcription factor, which promotes the expression of the BCL-2 inhibitor BIM in oocytes [79]. Treatment with KITLG inhibits FOXO3, BIM, and apoptosis in naked oocytes in vitro [80]. Thus, the shortage of available survival-stimulating pre-granulosa cells may translate to PGCs death until the optimal ratio between oocytes and pre-granulosa cells is achieved.

Growth factor deficiency, in general, promotes intrinsic apoptosis through the BCL-2- BAX/BAK axis. *Bcl2* or *Bax* knockout in mice results in decrease or increase in the number of primordial follicles, respectively [81, 82], and apoptotic oocytes display a reduced *Bcl2l1* to *Bax* transcripts ratio [83]. Independently of the PI3K/AKT and STAT3 pathways, DNA damage, ER stress, mitochondrial damage, or nutrient starvation can activate BH3-only proteins through p53 or its homolog TAp63; the latter protein is the major regulator of NOXA and BBC3 (PUMA) in oocytes [35, 63, 84]. BBC3, NOXA, and BAX are controlled by the checkpoint kinase 2 (CHK2)-p53/p63 checkpoint system and facilitate the elimination of primary oocytes with persistent DNA damage in the postnatal period [64, 65]. BBC3 seems to be more important for this process than NOXA: BBC3-only or BBC3/NOXA inactivation protects PGCs from radiation-induced apoptosis, but NOXA-only knockout does not [63]. Moreover, BBC3 is crucial for the death of PGCs during their migration to the developing ovary but not during PGC cyst breakdown; the second process seems to be BAX-independent [82, 85]. TAp63-dependent DNA damage and apoptosis in fetal oocytes are drastically promoted by the inactivation of Glycogen synthase kinase 3 beta (GSK3B) kinase [86]. This fact is surprising—as GSK3B activity is commonly suppressed by the PI3K/AKT pathway—and indicates the complexity of the interactions between pro-survival and pro-apoptotic signals in the establishment of the primordial follicle pool. DNA damage and repair are inherent to crossover recombination during meiosis, and the aforementioned information supports the idea of PGC depletion as a way to enrich the pool of competent oocytes that have not accumulated unrepaired DNA lesions.

Very little data are available regarding extrinsic apoptotic signals in the process of PGCs depletion. Primary oocytes express TNFα, TRAIL and their receptors, and treatment with TNFα or TRAIL in vitro can induce apoptosis in naked oocytes or granulosa cells, respectively [87, 88]. CD95/Fas/Apo-1 knockout increased PGC numbers in both fetal and postnatal murine ovaries [73]. Overall, this area requires further investigation.

The extrinsic and intrinsic apoptosis pathways converge on caspase machinery, and three distinct features have been reported for caspases in PGCs and primordial follicles. Caspase-3, one of essential executioners of apoptosis, seems to be completely irrelevant for oocyte pool dynamics in mice during both embryonic development and initial weeks after birth [89]. Caspase-2 is usually dispensable for apoptosis regulation [90], but *Casp2* knockout in mice results in a significant increase in the number of primordial oocytes [91]. Further investigation has demonstrated that caspase-2 deficiency prevents PGC death caused by cytokine deprivation (or inactivation of interleukin processing by caspase-4/-5 or caspase-11) but does not affect apoptosis induced by meiotic defects [92]. Selective caspase-2 inhibition is sufficient to partially prevent the death of oocytes cultured in vitro [69]. Initiator caspase-9 is constitutively active in all fetal oocytes, but the progression of apoptosis is hindered by the X-linked inhibitor of apoptosis (XIAP) protein [93, 94]. This block is relieved by upregulation of LINE1 retrotransposon expression [95]. It is possible that other caspases (caspase-6, caspase-7, and caspase-8) could also be regulated in a unique way in fetal oocytes, but no reports have been published on this topic.

Surprisingly, the hallmarks of classic apoptosis are not universally observed in murine primordial follicles during early postnatal development despite the ongoing reduction in oocyte numbers [13, 96]. This fact raises many questions about the contribution of apoptosis to the depletion of oocytes during the pre-puberty period.

### Autophagy

While apoptosis is the most investigated type of PCD in the embryonic ovary, some of the oocytes also display autophagic features both in vitro and in vivo [69, 97], but the current reports are as controversial as autophagy itself. A prominent increase in the number of LAMP1-labeled vesicles in oocytes soon after birth suggests that lysosomes and autophagy may be involved in the corresponding reduction of the oocyte pool [97]. However, LC3, which is essential for autophagosome maturation, is only detected in rodent PGC cysts and completely disappears from oocytes during primordial follicle formation; it is only retained in granulosa cells [98, 99].

Inactivation of autophagy by *Atg7* knockout or hemizygous *Becn1* deficiency in mice results in severe loss of PGCs during the neonatal period, suggesting its positive role in oocyte survival [100]. Germ cell-specific *Atg7* knockout causes the same effect, ruling out the involvement of granulosa cells [101]. Autophagy and the oocyte pool size are both positively controlled by miR-378-3p (which targets pyruvate dehydrogenase kinase 1 (PDK1) and caspase-9), miR-92b-3p (which targets tuberous sclerosis complex subunit 1 (TSC1), and *Sirtuin 1 *(*Sirt1)* [99, 102]. On the other hand, the lack of MAP1LC3 indicates that canonical autophagy should not be active in primordial follicle oocytes [98]. Therefore, it is possible that ATG7 or BECN1 deficiency confers their effects through a more exotic LC3-independent autophagic process. This idea is supported by the fact that oocytes in primordial and primary follicles contain cytoplasmic autophagosomes detectable by electron microscopy [103, 104]. An experimental model demonstrated that autophagy protects PGC cysts and oocytes from the stress caused by perinatal starvation, albeit at the expense of granulosa cells [103, 105].

Despite the observations described above, the promotion of autophagic process has been also reported during attrition of primordial follicles. Degrading oocytes in ovaries of 1- to 5-day-old rats express high levels of lysosome marker LAMP1 and acid phosphatase [104]. However, LAMP1 labels all kinds of lysosomes, including those produced in an autophagy-independent manner. In addition, some autophagic oocytes simultaneously display caspase-3 activation and DNA fragmentation without clear apoptotic morphology [104]. Therefore, the causative role of autophagy in oocyte death progression remains to be confirmed. Nevertheless, the idea of “altruistic death” has been proposed. The majority of PGCs in the pre-follicular cysts are considered to be “nurse cells” that sacrifice themselves to provide nutrition to one surviving oocyte [66]. In this case, ADCD would be a fitting way for the cells to die. Recent studies in *Drosophila* and mice have confirmed the idea of “nurse cells” but have proposed a unique model of extrinsic cell death induction instead of common autophagy [6, 67]. During follicle formation around the PGC cyst, surrounding pre-granulosa cells generate acidic extracellular vesicles that fuse with PGCs. Pre-granulosa cells transfer cathepsins and DNases into “nurse cells”, promoting their death and relocation of their contents into the developing oocyte through intercellular cytoplasmic bridges. This unique process is inhibited by bafilomycin A1, a commonly used inhibitor of lysosome generation and autophagy that blocks the activity of vacuolar ATPase and prevents acidification, resulting in PGC survival [6]. At first glance, such effects could be interpreted as evidence that autophagy is the reason for “nurse cell” degradation, but the real mechanism is very different because it is facilitated through acidified exosome-like vesicles instead of intra-cytoplasmic lysosomes. It is also possible that autophagosome-like structures observed in primordial follicle oocytes are transferred to their cytoplasm from “nurse cells”. More studies are needed to better understand this unique way of oocyte death.

### Necroptosis and ferroptosis

Necroptosis- or ferroptosis-mediated PCD in the developing ovary had not been investigated in detail until very recently. The fact that oocytes express TNFα was described back in 1994 [106], but the first evidence of necrotic cell death during the pre-puberty period was reported in 2018 [107]. Necroptotic ovarian cells comprise both PGCs and somatic cells immediately after birth, but only somatic cells still undergo necroptosis by day 21 [107, 108]. Surprisingly, the highest concentration of necroptotic events occurs in the medulla region, but its role is undefined [108].

Transferrin, a major iron carrier protein, is expressed at all stages of ovarian follicles and is used as an important component of medium for ovary cultivation conditions [109, 110]. Early stages of growing follicle atresia are associated with pro-ferroptotic changes in gene expression patterns [111], but there are currently no reports describing ferroptotic cell death events in the developing ovary. Nevertheless, a recent single-cell transcriptome study revealed that most ferroptosis-related genes are highly expressed in both oocytes and granulosa cells during the late embryonic stages and early postnatal life, sparking an increased interest in this area [112].

## Regulation of PCD during follicular atresia

Out of the thousands of oocytes that survive the initial PGC depletion and form primordial follicles, only a few are able to complete maturation and successfully ovulate [113]. The vast majority of follicles undergo spontaneous atresia at the antral stage unless they are rescued by FSH secreted by the pituitary gland [19]. The initial recruitment of primordial follicles begins around birth, but follicular atresia eliminates the growing follicles throughout the entire juvenile period. After puberty onset, periodic surges of gonadotropins during each estrous/menstrual cycle rescue a number of antral follicles from degradation and allow them to develop further. During this stage, one or more dominant follicles reciprocally suppress FSH secretion and thus exert an inhibitory effect upon other maturing ones, setting them toward atretic demise [22, 23].

**Fig. 3:**
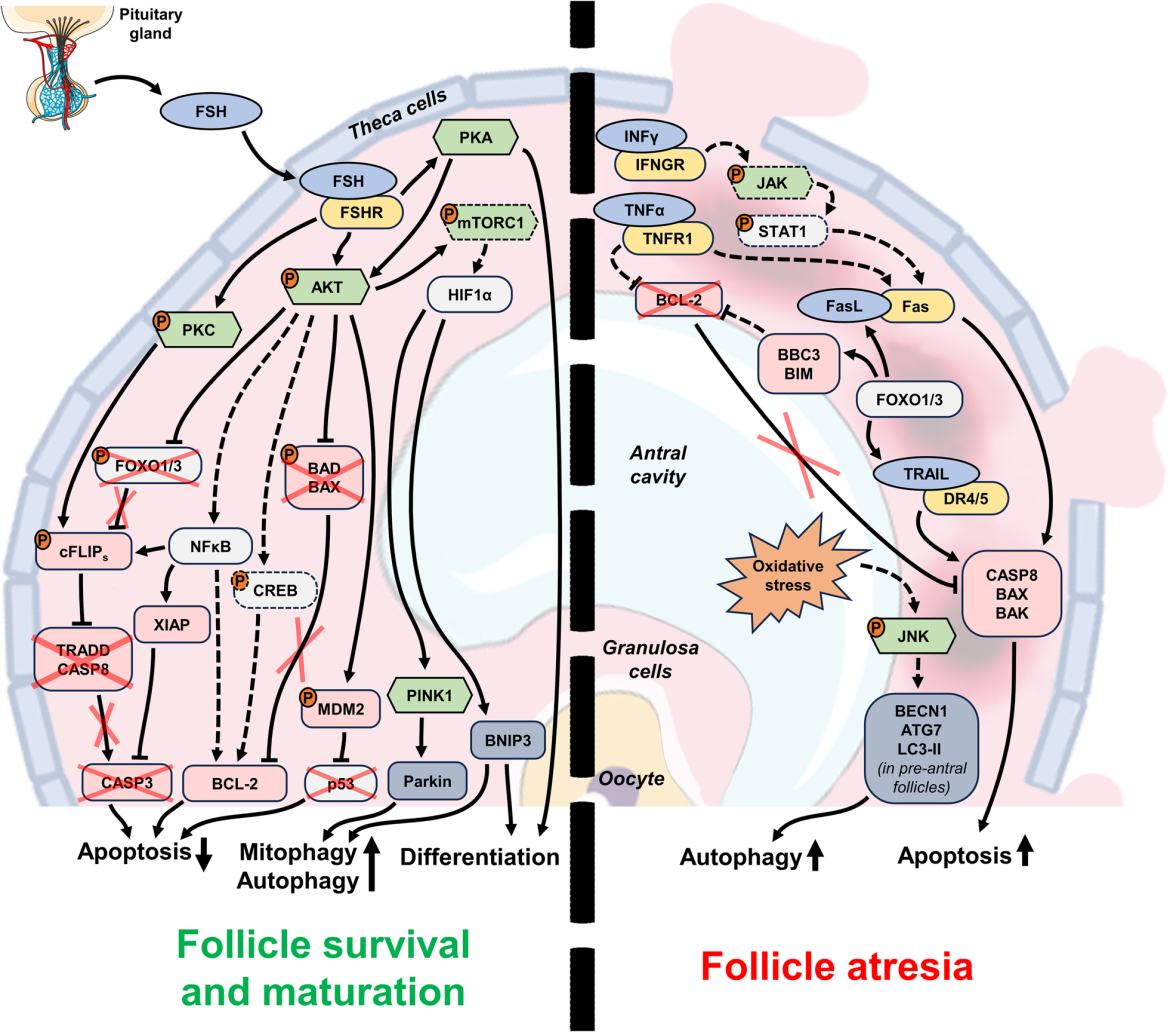
Major molecular mechanisms and regulatory elements involved in the control of antral follicle atresia in the cycling ovary. The pathways regulated by FSH, which is secreted by the pituitary gland, promote the survival of granulosa cells and the growth and maturation of the follicle (left side of the figure). These pathways inhibit apoptosis and promote pro-survival autophagy (and mitophagy in particular) in antral and dominant follicles. When FSH levels are insufficient, extrinsic apoptosis is induced in the granulosa cell layer and results in follicular atresia (right side of the figure). Pro-ADCD additionally promotes the degeneration of pre-antral follicles under the conditions of oxidative stress. Orange circles with the letter “P” indicate protein phosphorylation, red crosses indicate the lack or suppression of certain regulators, and dashed elements indicate the regulators and connections that are not definitively confirmed up to date

The major difference between PGC attrition during ovary development and atresia of antral follicles lies in the origin of degradation. While the depletion of primordial follicles is driven by oocytes themselves, follicular atresia is initiated in granulosa cells [114, 115]. Atresia can happen at any stage of follicular development, but early antral follicles are most susceptible to it [19]. Three types of atresia have been described based on where cell death is initiated: (I) “antral” atresia starts in the middle of the granulosa cell layer; (II) “basal” atresia spreads from the basal lamina and is associated with early incomplete luteinization of granulosa cells; and (III) “terminal differentiation” atresia occurs in preovulatory follicles, where globules of granulosa cells detach from the inner surface of the antral cavity and undergo apoptosis in the antral liquid [114, 116]. Despite differences in the initiation and progression details, the functional differences between these types of atresia are unclear. The initial focus of PCD quickly spreads over the granulosa cell layer and to the oocyte itself due to the presence of gap junction complexes that establish cytoplasmic bridges between cells. Such bridges may facilitate transfer of either nutrients from healthy cells or PCD mediators from atretic ones to the oocyte [20]. The most well-characterized regulators and signaling pathways of PCD involved in follicular atresia are depicted in Fig. 3.

### Apoptosis

Apoptosis seems to be a predominant type of PCD responsible for granulosa cell death and follicular atresia, and it is easily recognized by the above-mentioned features [115]. Unlike in the case of PGC depletion which is mostly dependent on intrinsic apoptosis, both intrinsic and extrinsic pathways are significantly involved in the control of follicular atresia. The extrinsic pathway activated by adjacent granulosa cells seems to constantly push them toward apoptosis, while pituitary hormones and growth factors rescue them from demise [115].

The CD95/Fas/Apo-1–FasL system is one of the most well-characterized regulatory pathways involved in atresia initiation. Both molecules are abundantly expressed in granulosa cells of atretic follicles in a pattern that closely follows the localization of apoptotic cells [117–119]. The levels of CD95/Fas/Apo-1 and FasL increase during the progression of atresia and correlate with both apoptotic activity in granulosa cells and gonadotropin exposure [120]. CD95/Fas/Apo-1 and FasL are expressed during all stages of follicle development starting at the pre-antral stage, but prominent apoptosis induction is not observed until the antral stage. This may be explained by a simple increase in the expression of CD95/Fas/Apo-1, FasL, or both [120], but may also be associated with the membrane localization of both CD95/Fas/Apo-1 and FasL [36]. In the latter case, pathway activation occurs upon achieving critical concentrations of both the receptor and ligand through an increase in cell density in the multilayer walls of the antral follicle.

While the oocyte does not initiate the process of atresia, it still expresses the components of the extrinsic apoptotic pathway. The data on CD95/Fas/Apo-1 and FasL in the oocytes are controversial. Some researchers have reported the expression of CD95/Fas/Apo-1 but not FasL [117, 121], but other investigators have detected both CD95/Fas/Apo-1 and FasL in oocytes at different stages of follicle development [118, 122]. Despite expressing CD95/Fas/Apo-1, the oocytes are not susceptible to FasL-dependent apoptotic stimuli exerted by granulosa cells because they are separated by the zona pellucida [19]. Removal of the zona pellucida layer promotes apoptosis in oocytes co-incubated with FasL-positive granulosa cells in vitro [117].

Treatment with CD95/Fas/Apo-1-activating antibody alone can promote granulosa cell death in vivo but not in vitro [123–126]. However, the susceptibility of granulosa cells to CD95/Fas/Apo-1 activation in vitro is greatly increased by treatment with Interferon-γ (IFNγ), TNFα or cycloheximide, indicating that some additional mechanisms are involved in the regulation of CD95/Fas/Apo-1 system [124–127]. IFNγ is expressed in both oocytes and granulosa cells of pre-antral and early antral follicles [127] and acts through JAK/STAT pathway [128]. IFNγ treatment results in a prominent increase in CD95/Fas/Apo-1 expression in granulosa cells, elevating their sensitivity to FasL and promoting apoptosis [125–127].

TNFα is a dualistic regulator of cell death and survival, and its various effects are executed through two receptors: TNFR1 (pro-apoptotic) and TNFR2 (pro-survival) [129]. All three molecules show moderate-to-high expression levels in all cells of pre-antral and antral follicles [130], but TNFα predominantly causes granulosa cell apoptosis and follicle degradation in various models like human granulosa cell culture [131], isolated rat antral follicles [132], and whole bovine ovarian tissue cultured in vitro [130]. Moreover, researchers have identified the TNFR1-mediated pathway as a potential upstream regulator of atresia in porcine follicles using a transcriptomic approach [111, 133]. Like INFγ, TNFα can induce overexpression of CD95/Fas/Apo-1 [126], but its effect can also be mediated through a decrease in BCL-2 protein level [131].

TRAIL is the third major regulator of extrinsic apoptosis, but its role in follicular atresia is poorly understood. TRAIL ligand, its functional death receptor (DR5), and the decoy non-functional (DcR1 and DcR2) receptors are expressed in growing and antral follicles; apoptosis is readily initiated in TRAIL-treated granulosa cells in vitro [88]. The detailed analysis of TRAIL signaling in porcine ovaries has demonstrated that healthy developing follicles express high levels of DcR1 that are lost in atretic granulosa cells, while DR4 levels remain unchanged [134, 135]. Moreover, inactivation of DcR1 in healthy granulosa cells results in their prominent sensitization to TRAIL [136]. On the other hand, the expression of TRAIL itself is prominently increased during follicle growth [135]. Unfortunately, this promising area presently feels very under-investigated.

FasL-, TNFα-, and TRAIL-dependent cell death is additionally regulated by a specific cFLIP protein that inhibits the formation of both pro-apoptotic and necroptotic complexes, death-inducing signaling complex (DISC) and ripoptosome, respectively. Three common isoforms of cFLIP (cFLIP_L_, cFLIP_S_, and cFLIP_R_) have been described, and cFLIP_S_ and cFLIP_R_ are considered to play a more important role in RCD control due to stricter regulation of their abundance in cells [137]. The published studies on cFLIP in ovarian follicles have mostly used the model of porcine ovaries, where it is prominently expressed in granulosa cells during follicle growth [138]. Follicular atresia is associated with a reduction in cFLIP_L_ but not cFLIP_S_ transcription; on the other hand, cFLIP_S_ protein is not detected in any condition [139]. Overexpression or suppression of cFLIP variants in cultured human and porcine granulosa cells renders them resistant or sensitive to CD95/Fas/Apo-1-induced apoptosis, respectively [140]. Furthermore, cFLIP controls granulosa cell death through caspase-8, confirming a unique function for cFLIP in extrinsic apoptosis [141]. Despite the well-established role of cFLIP in apoptosis, its regulation is not fully characterized. The half-life of cFLIP is very short [142], and this fact explains prominent sensitization to CD95/Fas/Apo-1-induced apoptosis by cycloheximide, as general translation inhibition quickly depletes the cFLIP supply [126]. Protein kinase C (PKC)-dependent phosphorylation of cFLIP_S_ stabilizes the protein and prevents its rapid degradation [143], while its expression is stimulated through the AKT-FOXO3 and AKT-NFκB pathways [142, 144]. Both PKC and AKT pathways are stimulated by FSH (see below), adding cFLIP as an impactful factor in overcoming PCD in the antral follicle.

Modulation of the intrinsic pathway components in antral follicles is mostly associated with the maintenance of cell survival. Due to the developed net of blood vessels, growth factor shortage is rarely a problem in the mature ovary, but additional stimuli are required to prevent extrinsic apoptosis. FSH is the main factor that prevents follicles from inevitable apoptotic demise [19]. It is indispensable for the formation of antral follicles, as genetic knockout of either the β-subunit of FSH or the FSH receptor (FSHR) results in the complete absence of antral follicles, even atretic ones, in murine ovaries [145, 146]. It is also crucially important for the survival of granulosa cells. The peptide inhibitor of the FSH-FSHR interaction causes follicular atresia in rodents and primates [147, 148], while an excess of FSH prevents apoptosis in dominant follicles [149]. FSHR is expressed on the surface of granulosa cells in pre-antral and antral follicles [21], and its activation affects a plethora of intracellular signaling pathways through G proteins and β-arrestins, multifunctional scaffold proteins [150, 151]. The major regulatory circuits activated by FSH include the cAMP/PKA, PI3K/AKT, PLC/PKC, and MEK/ERK pathways. Current knowledge suggests that G proteins provide a rapid response via all of these pathways, while β-arrestins are involved in prolonged stimulation of only MEK/ERK signaling [150, 152]. These signaling pathways are tightly interconnected, but PI3K/AKT and cAMP/PKA seem to be the most important for granulosa cell survival and further follicle maturation, respectively [150, 153]. The experimental data indicate that cAMP/PKA-regulated granulosa cell survival may be mediated through AKT stimulation, and the differentiation-related effects of PKA are independent of PI3K/AKT [154, 155]. Differentiation and terminal maturation of antral follicles curated by cAMP/PKA are controlled through steroid hormone synthesis [156]; however, these processes lie beyond the scope of our discussion.

The PI3K/AKT pathway plays an important role in follicular atresia as it controls a multitude of apoptotic regulators [157]. It confers general anti-apoptotic effects mediated through upregulation of BCL-2 and suppression of BIM [77, 158, 159], inactivation of BAX and BAD [78, 160], and activation of the p53-inhibiting mouse double minute 2 homolog (MDM2) protein [77]. Furthermore, the FSH-stimulated AKT pathway promotes the expression of XIAP that inhibits the activity of effector caspases, preventing the induction of apoptosis through the intrinsic and extrinsic pathways [161, 162]. Atretic follicles display a decrease in XIAP compared with healthy antral follicles [163]. AKT can increase the XIAP level through NFκB-mediated stimulation of its transcription [164], and XIAP has been proposed as a major switch between follicle survival and atresia [165]. However, the pivotal role of XIAP can be challenged by FOXO transcription factors (FOXO1 and FOXO3) that are widely known as direct targets of the AKT pathway. FOXO proteins promote apoptosis through transcriptional upregulation of multiple pro-apoptotic genes. For example, FOXO1 facilitates overexpression of BBC3 and apoptosis in response to oxidative stress in murine granulosa cells [166, 167]. FOXO1 expression is drastically increased in early antral follicles; it is suppressed again in preovulatory follicles after gonadotropin stimulation but persists in atretic ones [168]. There is a similar trend, albeit with less prominent changes, for FOXO3 [169]. More importantly, FOXO transcription factors promote the expression of FasL and TRAIL [120, 169], which, as mentioned above, are directly involved in granulosa cell atresia [170]. This fact has been confirmed experimentally in porcine ovaries [169]. FOXO phosphorylation by AKT results in protein translocation from the nucleus to the cytoplasm and subsequent degradation [170]. Therefore, it is logical to assume that FSH-dependent rescue of antral follicles is mediated through the AKT-FOXO axis and results in FasL and TRAIL downregulation [155]. However, FSH treatment does not affect FasL expression in granulosa cells [149]. Surprisingly, simultaneous knockout of the *Foxo1* and *Foxo3* genes in granulosa cells results in a drastic reduction in the serum FSH level, indicating that there is a regulatory feedback loop between maturing ovarian follicles and the pituitary gland [171]. A recent proteomic analysis of atretic granulosa cells identified FOXO1 and AKT3 among the top regulators of follicular atresia in pigs [172]. Thus, PI3K/AKT signaling affects both extrinsic and intrinsic apoptosis in antral follicles, FSH synthesis and dominant follicle selection. Researchers should pay much attention to this pathway when exploring the molecular mechanisms of follicular atresia.

While FSH is the main factor regulating follicle survival, other molecules contribute to this process, including IGF, the transforming growth factor (TGF) superfamily, Epidermal Growth Factor ( EGF), Fibroblast Growth Factor (FGF), estradiol, interleukins, and other regulators. Many of them (IGF, EGF, and bFGF) exert their effects through the same PI3K/AKT and MEK/ERK pathways, augmenting FSH-dependent effects [173–176]. IGF is especially interesting because it is required for proper activation of the PI3K pathway by FSH [177]. The TGF superfamily comprises a multitude of different proteins, of which activin and inhibin are the most relevant due to their ability to induce or suppress FSH secretion, respectively. The effects of TGF receptors activation are facilitated through SMAD proteins (the main signal transducers for receptors of the TGFβ superfamily) and ultimately result in increased FSHR expression [152]. The functions of estradiol and interleukins in follicular atresia are not completely understood. Studies in rodents and cattle suggest that estradiol should promote granulosa cell survival via BCL-2 regulation; however, the experiments performed on primates demonstrate that estradiol can induce atresia of dominant preovulatory follicles [178]. Interleukins control multiple regulatory circuits. Although their effects may be very controversial, the accumulated data imply that their actions are cytoprotective rather than pro-apoptotic [179].

The final stages of apoptosis in atretic follicles significantly depend on activation of caspase-3, in contrast to caspase-3-independent apoptosis in primordial follicles [89]. The pattern of caspase-3 activation in the normal ovary overlaps with the localization of atretic foci [180]. However, caspase-3 depletion is not sufficient for complete prevention of follicular degradation, as caspase-7 and caspase-9 are also involved in the process and can compensate for caspase-3 loss [89, 181]. Overall, terminal execution of apoptosis in the atretic follicles exhibits much more “canonical” mechanisms than in PGCs and primordial follicles.

### Autophagy

The role of autophagy during follicle growth and atresia in the adult ovary is even more diverse and controversial than during embryonic development. The first mention of autophagy in atretic granulosa cells of quail ovaries was in 1996 [182]. However, ADCD in the follicles of human ovaries was first described in 2006, and this opened a new direction in studies of follicular atresia [183].

The first report of localization of the crucial autophagic regulator BECN1 in a normal ovary suggested its exclusive expression in theca cells, and not in granulosa cells or oocytes [184]. Nevertheless, later reports demonstrated that granulosa cells express BECN1, as well as ATG5, ATG7, MAP1LC3, and other autophagy-related proteins, which indicates that these cells are competent for autophagy [185–187]. While researchers initially thought that autophagy occurs in follicles in the context of the stress response to oxidized low-density lipoproteins [183], they soon discovered that antral follicle atresia is associated with active autophagy under normal physiologic conditions [188]. This finding was supported by the fact that oxidative stress is one of the factors promoting death of granulosa cells [189]. However, starting from this point, the reports describing the effects and regulation of autophagy in the follicles become very inconsistent and mainly follow one of two contradictory concepts.

The first concept regards autophagy in follicular atresia as a destructive cell death process. A comparative investigation of autophagic regulators and active caspase-3 expression in different types of follicles suggested that atresia of pre-antral follicles is executed through autophagy, while late antral follicles degrade via apoptosis [190]. Pre-antral follicles are extremely dependent on the presence of FSH, and, based on the current knowledge of FSH-dependent signaling, it should inhibit autophagy (alongside apoptosis) via the PI3K/AKT/mTORC1 pathway [41]. Several studies have confirmed this idea, reporting AKT pathway activation, LC3-II downregulation, and a reduction in the number of autophagosomes after gonadotropin treatment in vitro or in vivo, while PI3K, AKT, or mTORC1 inhibitors prevent FSH-dependent effects [188, 191]. FSH-associated inhibition of mitophagy is even able to protect granulosa cells from oxidative stress [192]. Oxidative stress can promote autophagy through JNK activation; however, the exact mechanism in granulosa cells remains vague [193, 194]. It was demonstrated that autophagy activation during oxidative stress precedes apoptosis, and *ATG7* or *BECN1* knockdown rescues cells, confirming that the observed phenomenon can be correctly considered ADCD [195].

On the other hand, a growing number of reports support an alternative concept concerning autophagy-mediated protection of granulosa cells from death. This idea is consistent with the common knowledge of autophagy acting as a survival mechanism in stressful conditions [39]. *Epg5* gene knockout suppresses autophagy and leads to accumulation of WT1 and subsequent induction of apoptosis in granulosa cells [196]. Alternatively, the autophagic response can be induced in hypoxic conditions through Hypoxia Inducible Factor 1 Subunit Alpha (HIF1α) and is involved in a metabolic switch to glycolysis [197]. Surprisingly, both HIF1α and autophagy are stimulated in hypoxic murine granulosa cells by FSH treatment. Furthermore, FSH treatment somehow inhibits mTORC1 phosphorylation without inhibiting AKT [198]. These facts have been confirmed in porcine cells and attributed to mitophagy regulated through the HIF1α-PINK1-Parkin cascade [198, 199]. The regulatory circuits connecting FSH, the PI3K/AKT/mTORC1 pathway, and autophagy in granulosa cells may be heavily dependent on oxygen balance. FSH-PI3K/AKT signaling always acts as an anti-apoptotic factor, but it has to either compete with destructive autophagy (during oxidative stress response) or cooperate with protective mitophagy (in hypoxic conditions). Later studies revealed that HIF1α-BNIP3-stimulated autophagy promotes granulosa cell survival and differentiation into luteal cells [200, 201]. miRNA-21-3p can simultaneously inhibit VEGFA, FGF2, the AKT/mTORC1 pathway, and autophagy in bovine granulosa cells [202, 203], raising even more questions about the connections between membrane receptors, the PI3K/AKT/mTORC1 pathway, and autophagy in granulosa cells.

While it is well-established that follicular atresia begins in the granulosa, the mechanisms of oocyte degradation after breakdown of the granulosa layer are mostly unknown. The deterioration of cytoplasmic protrusions that sprout through the zona pellucida from granulosa cells should result in a drastic reduction of nutrients transported to the oocyte, and it is no surprise that the oocytes in the atretic follicles display clear signs of autophagy. Their cytoplasm is filled with a high number of autophagosomes and displays prominent LAMP1 and MAP1LC3 staining [104, 204]. Simultaneously, the same oocytes are positive for cleaved caspase-3 and DNA fragmentation, two hallmarks of apoptosis [104, 204]. The seemingly unique feature of atretic oocytes is the disturbance of cytoplasmic structures known as lamellae or lattices which could also be attributed to ongoing autophagy [205]. It is still unknown whether autophagy plays a driving role in oocyte degradation or just coincides with apoptosis in a futile attempt to rescue the stressed oocyte. The only available evidence of two processes being possibly connected is provided by protein-protein interactions between BECN1 and BAX, BCL-2, and cleaved caspase-3 [206], but no functional studies have been published.

### Necroptosis and ferroptosis

The role of necroptosis and ferroptosis in follicular atresia was not properly addressed until very recently. These two types of cell death have not been detected consistently in normal ovaries in vivo, but there is recent evidence that they can contribute to granulosa and oocyte death.

The first evidence of necroptotic death in granulosa cells was reported in a study focused on a readthrough isoform of acetylcholinesterase (AChE-R) in ovarian follicles. A synthetic peptide derived from the AChE-R C-terminal region induced MLKL phosphorylation and necroptosis in cultured granulosa cells [207]. Researchers confirmed the ability of AChE to regulate MLKL phosphorylation (seemingly through RIPK1) by using a three-dimensional in vitro culture of macaque follicles, but no associations between necroptotic proteins and follicle degradation were detected [208]. The *RIPK1* and *RIPK3* genes are overexpressed in atretic bovine follicles, and a system biology approach suggested that necroptosis may be actively involved in atresia [209]. SIRT1 promotes granulosa cell death and regulates RIPK1 and MLKL phosphorylation; however, the mechanism behind cell death is RIPK1-dependent apoptosis rather than MLKL-dependent necroptosis because it is accompanied by caspase-3 processing and PARP cleavage [210].

Transcriptomic analysis of early atretic follicles in a pig ovary revealed that the gene expression changes during atresia initiation represent a crosstalk between apoptosis, autophagy, and ferroptosis rather than apoptosis alone (including disturbances in GSH metabolism) [111]. This is consistent with the ultrastructural changes described in early atretic oocytes that include mitochondria condensation and loss of cristae—classic signs of ferroptosis [211]. Inactivation of the basonuclin zinc finger protein 1 (*BNC1)* gene promotes ferroptotic oocyte death through the neurofibromin 2 (NF2)-Yes associated protein (YAP) pathway [212]. Ferroptosis contributes to granulosa cell death in patients with endometriosis through  the accumulation of iron in the follicular liquid, confirming the importance of iron balance for follicle survival [213]. The circRHBG-miR-515-5p-SLC7A11 molecular axis, which is often upregulated in patients with polycystic ovary syndrome (PCOS), inhibits ferroptosis in granulosa cells [214]. More investigations are required to determine whether ferroptosis is a normal event during follicular atresia in the healthy ovary.

The available data suggests that cells of antral follicles have the required molecular machinery to undergo necroptosis or ferroptosis; however, these types of RCD seem to represent minor variants in comparison to apoptosis and autophagy. Both necroptosis and ferroptosis have been known for more than a decade, but the scarce mentions of them in the recent literature imply that their characteristic features are rarely observed outside of specific circumstances (AChE-R overexpression, *BNC1* mutation, etc.). Theoretically, FasL- or TRAIL-induced necroptotic death could occur in cells with inactivated cFLIP_L_ when apoptotic pathways are suppressed by FSH signaling, but this hypothetical event has to be confirmed experimentally in vitro and in vivo. Ferroptosis is inhibited by PI3K/AKT signaling [215] and can be promoted by autophagic degradation of ferritin [216]. Therefore, it could be prone to occur in combination with autophagy when pre-antral follicles receive insufficient FSH. While the proposed situations look rather exotic, a meticulous researcher should keep these mechanisms in mind when analyzing follicular atresia.

## Regulation of PCD during luteolysis

The CL is a temporary gland that exists in a tight reciprocal conjunction with the newly formed embryo. Progesterone secreted by the CL provides a suitable environment for embryo implantation and development in the uterus. After successful implantation, the uterus and the embryo start secreting CG or prolactin that maintain the CL in a functional state until the placenta is developed enough to support the embryo on its own [26, 27, 29]. Hence, it is extremely important to have a precise regulatory system that ensures rapid CL regression in case of fertilization or implantation failure so that the ovary can enter a new menstrual/estrous cycle.

**Fig. 4:**
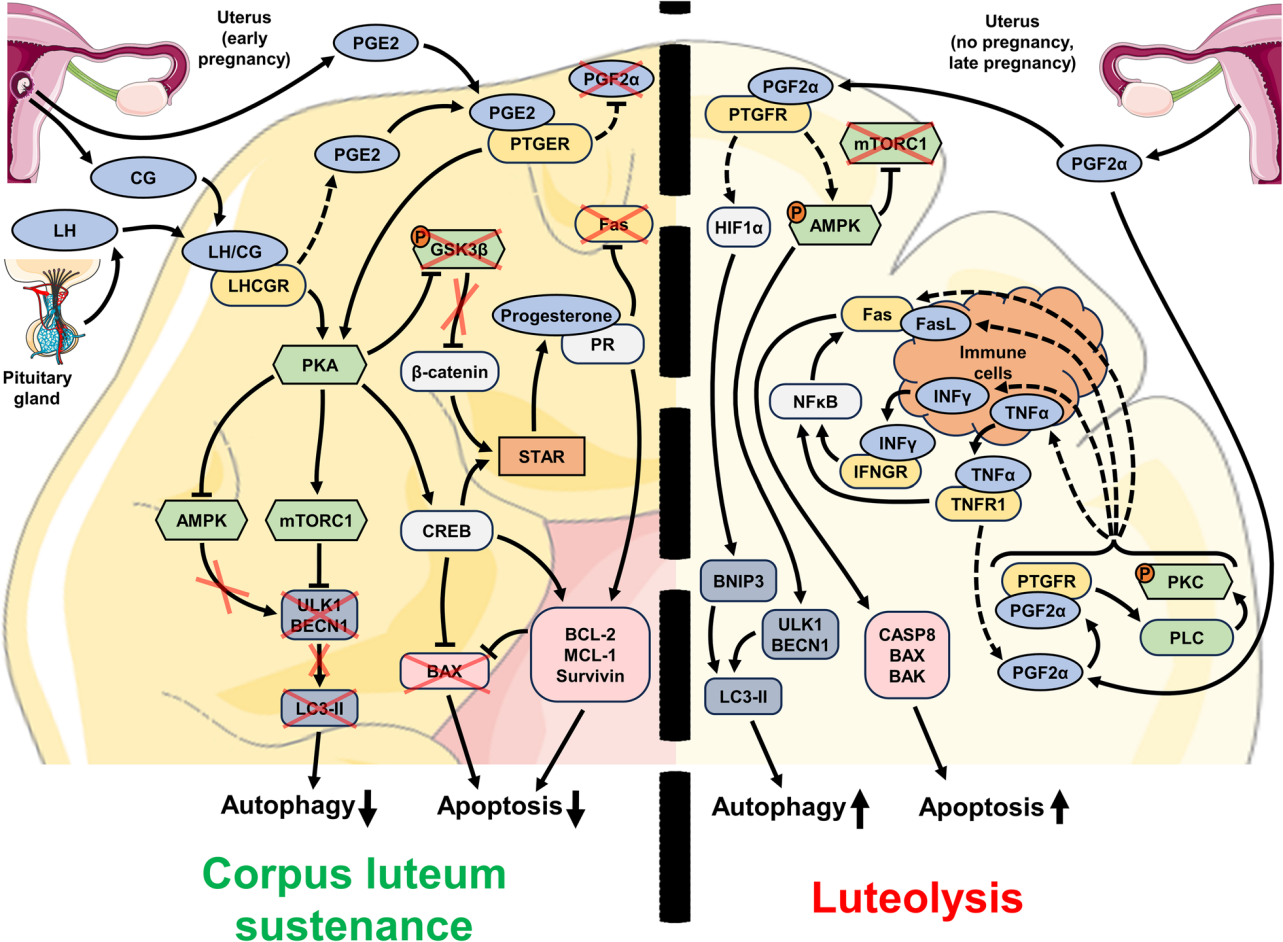
Major molecular mechanisms and regulatory elements involved in the control of luteolysis in the cycling ovary. LH secreted by the pituitary gland and CG secreted by the uterus during the early pregnancy period ensure maintenance of CL  and continuous synthesis of progesterone. Signaling pathways controlled by LHCGR-PKA regulatory axis suppress apoptosis and autophagy until the levels of LH and CG decrease (left side of the figure). If the pregnancy did not occur or is at late stages, then the uterus produces PGF2α, which initiates luteolysis through the promotion of extrinsic apoptosis and destructive autophagy (right side of the figure). The induction of apoptosis through death receptors is dependent on the presence of immune cells which express FasL. Orange circles with the letter “P” indicate protein phosphorylation, red crosses indicate the lack or suppression of certain regulators, and dashed elements indicate the regulators and connections that are not definitively confirmed up to date

The mechanisms of luteolysis are currently less understood compared with PGC depletion and follicular atresia. One of the major reasons for this issue is that different orders of mammals have different mechanisms of luteolysis regulation. For example, the majority of animals utilize uterus-derived PGF2α as the leading stimulator of CL regression, but primates (including humans) demonstrate successful luteolysis even after hysterectomy [27]. Conversely, primates have a unique hCG hormone that is secreted by trophoblast of the developing embryo and delays luteolysis. In other animals these functions are performed by LH, prolactin, or placental lactogen [26, 29]. Nevertheless, the aggregated knowledge establishes that luteolysis is kept in the balance by external inductors of cell death and survival (Fig. 4).

### Apoptosis

Similarly to the other cases of massive PCD in the ovary, apoptosis is the best studied mechanism of CL degeneration due to the long history of observations [217, 218]. A massive increase in apoptotic activity occurs in the CL tissue soon after ovulation if the ovum is not successfully fertilized [218]. Experimental abrogation of apoptosis, which is commonly performed by caspase-3 knockout, is complicated in the case of the CL due to prominent perinatal mortality of caspase-3-deficient mice [219]. However, in the cohort of mice that survived long enough, caspase-3 knockout prevented apoptosis progression in the ovaries and significantly hindered CL regression [220, 221]. CL development in the ovary is associated with active angiogenesis [222]; therefore, it is unlikely that the lack of nutrients or hypoxia could be the main reason for the induction of luteal cell death. It is currently considered that extrinsic apoptosis in CL is activated by a local increase in CD95/Fas/Apo-1 system components (with additional influence from IFNγ and TNFα) and by external PGF2α secretion [217].

Both CD95/Fas/Apo-1 and FasL are expressed in luteal cells in various model organisms in the case of a normal estrous/menstrual cycle or pregnancy [119, 123, 125, 223–225]. The levels of CD95/Fas/Apo-1 seem to increase progressively with the age of the CL [223–227]. CD95/Fas/Apo-1 and FasL are expressed in CL cells in a mutually exclusive way and cooperate for efficient apoptosis induction. CD95/Fas/Apo-1 is only present in steroidogenic luteal cells, while FasL is specific to immune cells (macrophages and T lymphocytes) that invade CL during the functional degradation stage and promote structural involution [228, 229]. However, this model of luteolysis needs confirmation from different sources. Activation of CD95/Fas/Apo-1 by a specific antibody or FasL results in luteal cell death in vitro and in vivo [123, 125, 224]; accordingly, mice with an inactivating FasL mutation display impaired luteolysis [123]. A specific mouse strain known as the SAMP (senescence-accelerated mouse-prone) displays many early signs of aging including a reduced reproductive potential due to the abnormal accumulation of luteal bodies in the ovaries. These CL-like formations do not undergo luteolysis and display a simultaneous reduction in CD95/Fas/Apo-1, FasL, and active caspase-3 levels [230].

The susceptibility of luteal cells to CD95/Fas/Apo-1-dependent apoptosis is further promoted by TNFα and IFNγ [125, 224, 226, 229, 231]. Studies of murine ovaries suggest that TNFα may always be expressed in CL tissue and sustains a baseline level of CD95/Fas/Apo-1-mediated death; however, a local increase in the IFNγ level drives prominent apoptosis induction [232]. On the other hand, TNFR1-deficient mice or mice treated with TNF-inactivating antibody display disturbances of the estrous cycle: they often remain in diestrus, probably due to impaired luteolysis [233, 234]. Both TNFα and IFNγ increase the expression of CD95/Fas/Apo-1, therefore, enhancing the apoptosis-inducing effects [125, 224, 232]. The exact mechanism of this regulation in luteal cells has not yet been determined, but the studies performed in other models suggest that it may be facilitated through NFκB-RelA-dependent control of transcription [235, 236]. Additional mechanisms of CD95/Fas/Apo-1-dependent regulation of apoptosis may include suppression of the soluble anti-apoptotic FasB decoy receptor and TGFβ1 [232, 237]. In general, the CD95/Fas/Apo-1-dependent mechanism of luteal cell apoptosis is very similar to the mechanism observed in atretic follicles (see “Regulation of PCD during follicular atresia - Apoptosis” section).

PGF2α has long been known as a luteolysis inductor that is external to the ovary itself. It is well established that PGF2a is secreted by the uterus and is crucial for CL regression in many species, with the notable exception of primates. Hysterectomy does not affect the CL lifespan in primates, but the administration of exogenous PGF2α still promotes CL degradation [27]. Many aspects of PGF2α functions remain poorly understood. For example, the ideas about the intraovarian source of PGF2α were proposed in the early 1970s [238] and were experimentally confirmed in 1976 [239], but the dynamics of prostaglandin synthesis in luteal cells were only determined in 2000 [240]. The most peculiar detail is that the regulatory mechanisms of PGF2α synthesis in the late-phase CL seem to establish a positive feedback loop, resulting in self-sustaining secretion of this major luteolytic factor [241].

The molecular pathways involved in signal transduction from the PGF2α receptor (PTGFR) to apoptotic regulators are also unclear. The only confirmed regulatory circuit associated with PTGRF is the PLC/PKC pathway, but its connection to cell death induction in the CL has to be further investigated [242, 243]. No interactions between PTGFR and extrinsic cell death regulators like FADD, TRADD, or caspase-8 have been identified; hence, it is very unlikely that PGF2α can directly induce apoptosis or necroptosis. Caspase-3-deficient mice that survive long enough to develop the CL in the ovaries are insensitive to the luteolysis-inducing effect of PGF2α, but they do demonstrate a drastic increase in caspase-8 activation upon treatment with either a Fas-activating antibody or PGF2α [221]. Moreover, PGF2α treatment increases the expression of CD95/Fas/Apo-1, FasL, accumulation of cleaved BID, and rise in BAX/BCL-2 ratio, while caspase-8 inhibition rescues cells from apoptosis, confirming that PGF2α promotes PCD by stimulating the extrinsic apoptotic pathway [244]. Injection of the PGF2α analog cloprostenol results in a significant increase in CD95/Fas/Apo-1, FasL, IFNγ, and TNFα expression within 4–12 h, suggesting strong upregulation of the extrinsic apoptotic pathway [245]. At the same time, IFNγ and TNFα reciprocally promote PGF2α secretion in luteal cells, closing the positive autoregulation circle [246]. In addition, PGF2α-induced CL regression is associated with PI3K/AKT pathway inhibition, STAT3 activation, ROS generation, and ER stress, indicating that the intrinsic pathway is at least partially involved in the sensitization of luteal cells to apoptosis [247–250]. Despite these aforementioned details, our understanding of PGF2α-dependent mechanisms of luteolysis is still far from complete. Nevertheless, we can be confident that the CD95/Fas/Apo-1-TNFα-IFNγ-PGF2α network is a cornerstone of cell death induction in CL.

In the majority of tissues, the final steps of apoptosis in the CL are mostly controlled by activation of caspase-3 [220, 221]. However, caspase-2 presents the most prominent changes in the late-stage CL, displaying a more than sevenfold increase in activity, while the relative activation of caspase-9 and caspase-3 is less prominent [251]. Unfortunately, there are no detailed reports on special features of caspase-2 in the CL, and this aspect of luteolysis regulation deserves more attention in the future.

Similarly to antral follicles that are inclined to CD95/Fas/Apo-1-dependent apoptosis, the CL seems to be predisposed toward luteolysis at the end of the menstrual/estrous cycle. Therefore, the impending apoptosis of luteal cells should be averted for successful embryo development. The onset of pregnancy is associated with the secretion of signaling factors aimed at preserving the CL and further stimulating its progesterone secretion. These factors are secreted by the developing embryo itself, associated membranes, the placenta, or the uterus at the location of embryo implantation and include CG (in primates only), prostaglandin E2 (PGE2), prolactin, and placental lactogen (in rodents). To some extent, the CL is also sustained by LH secreted by the pituitary gland [26, 252, 253].  The most important effects seem to be mediated by CG/LH and PGE2.

LH and CG are the primary stimulators of CL maintenance during pregnancy in ruminants and primates, respectively [26]. They act through the same LHCGR receptor that is expressed in the CL during all stages of the menstrual/estrous cycle or pregnancy except for the involution stage [254]. LHCGR is a G protein-coupled receptor that is capable of independent interaction with two separate G proteins thus mediating activation of either PLC/PKC, cAMP/PKA, or both pathways [255]. While the cAMP/PKA pathway is responsible for steroidogenic changes and progesterone secretion and acts through CREB- and GSK3β/β-catenin-dependent regulation of STAR enzyme expression [156, 256–258], PLC/PKC signaling may be associated with antisteroidogenic effects [259]. The possible effects of LHCGR-dependent PLC/PKC activation on luteal cell survival are unclear, especially considering that luteolytic PGF2α also activates this pathway [242]. A more exotic LHCGR partner is β-arrestin that may switch the downstream signaling toward AKT and MAPK cascade stimulation (including the pro-survival MEK/ERK pathway) [255, 260, 261].

LH or CG treatment promotes the viability of luteinized granulosa or luteal cells in vitro [231, 262–264] and protects the CL from regression in vivo [265–267]. The molecular changes that link LHCGR activation to anti-apoptotic effects include upregulation of BCL-2 [262, 267, 268], MCL-1 [263], survivin [269], and anti-oxidant enzymes [264] with simultaneous suppression of BAX and CD95/Fas/Apo-1 [262, 268]. The exact mechanisms facilitating these LH/CG effects in the luteal cells are still undetermined, but they may be mediated through PKA-CREB regulation of gene expression [258]. It is noteworthy that progesterone, the main product of luteal cells stimulated by LH or CG, suppresses luteolysis, reduces CD95/Fas/Apo-1 expression, and increases the BCL-2 level [270–273]. LHCGR stimulation is also associated with an increase in intraovarian PGE2 [265], the most important competitor of PGF2α in terms of defining the fate of the CL [274].

PGE2 acts through four different receptors (PTGER) that facilitate signal transduction through the cAMP/PKA pathway; PTGER2 and PTGER4, which are expressed in luteal cells, activate PKA signaling [274, 275]. PGE2 synthesis intensifies in the uterus after embryo implantation, and PGE2 is subsequently transported to the ovaries [275]. Researchers suggest that the initial PGE2 effects exerted upon the freshly developed CL define luteal cell sustenance before LH/CG influence; however, the actual relationships between PGE2 and CG/LH are unknown [276]. PGE2 on its own exerts anti-apoptotic effect [273], and its withdrawal from luteal cells results in activation of PGF2α secretion and shift toward luteolysis [277]. The current knowledge of apoptosis regulation during luteolysis is clearly incomplete, especially compared with well-defined mechanisms controlling embryonic oocyte attrition and follicular atresia. While the accumulated studies have established that the CL fate is defined by antagonism between the CD95/Fas/Apo-1-TNFα-IFNγ-PGF2α and the LH/CG-PGE2-PKA signaling networks, much more investigation is required in this area to clarify the details.

### Autophagy

The CL is a relatively large structure, and its degradation should result in large amounts of cellular material being discharged. Therefore, it is no surprise that autophagy, which actively recycles cellular contents, is prominent in luteal cells, especially during the menopausal or post-pregnancy involution [278, 279]. A comparative study of CL tissue morphology during luteolysis suggested that autophagy may be the predominant mechanism during the late stages of regression [280]. Nevertheless, the authors did not use specific autophagy-associated markers to make this conclusion definitive.

All key autophagic regulators (BECN1, MAP1LC3, ATG proteins, and LAMP1) are expressed in luteal cells [281, 282], and their abundance changes according to luteolysis dynamics. The MAP1LC3, LC3-II, LAMP1, and ATG family protein levels increase progressively during the luteal/diestrus stage and reach their maxima in the late-stage steroidogenic cells [248, 281–284]; a similar trend is observed in the CL during pregnancy [285]. However, LAMP2 levels peak at the mid-luteal stage and then decrease, indicating that autophagy during late luteolysis may be abrogated at the autophagolysosome formation step despite prominent initial induction [285]. The data on BECN1 are conflicting, as its expression seems to be the highest in the late-stage CL in pigs [281] and almost absent at the same time in humans [184]. The aggregated data suggest that moderate autophagic activity may be associated with luteal cell differentiation and survival during CL formation, but its prominent stimulation during the late luteal/diestrus phase is likely to contribute to tissue degeneration and PCD.

Luteal cells treated with PGF2α in vitro and in vivo display prominent signs of both autophagy and apoptosis [248, 283, 286]. While PGF2α-dependent apoptosis is supposedly associated with the PLC/PKC and the CD95/Fas/Apo-1–FasL pathways, autophagy stimulation may be facilitated through AMPK signaling activation in bovine [286], ER stress in goat [248], or HIF1α-BNIP3-dependent ROS generation in the rat CL [287]. The last observation is especially interesting as activation of the same pathway in granulosa follicle cells results in their survival and luteinization instead of PCD [200, 201]. Moreover, autophagy stimulation by PGF2α in rat luteal cells is independent of mTORC1 activity and is controlled through the MEK/ERK pathway; however, the possible connection between the MEK/ERK pathway and PTGFR is unknown [288]. In contrast, LH promotes PKA and mTORC1 signaling pathways and inhibits autophagy in bovine CL [286].

Autophagy activation in the late-stage CL is accompanied by caspase-3 cleavage and apoptosis [282–284]. Inhibition of autophagosome formation with 3-methyladenine rescues apoptotic cells, confirming that autophagy can be the driving force behind luteal cell death. Surprisingly, the administration of the common autophagy inhibitors bafilomycin A1 or chloroquine does not replicate the effects of 3-methyladenine, promoting apoptosis instead. The main difference between these treatments is that bafilomycin A1 and chloroquine do not inhibit autophagy initiation but prevent autophagosome disposal; as a result, the accumulation of immature autophagosomes may be a driving factor of ADCD during late luteolysis [248, 283, 285]. Similarly to follicular atresia, the main challenge in investigating autophagy in CL degeneration lies in discerning autophagy-driven apoptosis from the co-occurrence of apoptotic and autophagic processes.

### Necroptosis

Involution of the CL is a relatively “clean” process of tissue degradation that seems to take place without pronounced inflammation, which is one of the distinct necrosis/necroptosis features. Nevertheless, luteolysis strongly depends on CD95/Fas/Apo-1-, FasL-, and TNFα-dependent signals which are also involved in necroptosis. This idea is supported by the fact that luteal cells express significant levels of AChE and phosphorylated MLKL, and MLKL phosphorylation is increased in the late-stage CL [207, 289].

Clear necroptotic events have not been described in the normal CL. Two reports refer to an increase in *RIPK1* and *RIPK3* expression and cell death induction in the bovine CL and cultured luteal cells in response to PGF2α, TNFα, and IFNγ treatment [290, 291]. However, neither study includes data on RIPK1 or RIPK3 phosphorylation/activation or MLKL changes. They also only utilize RIPK1 inhibitor necrostatin-1 as a functional confirmation of necroptosis, which is not sufficient [47]. Considering that the authors demonstrated caspase-3 activation in the dying cells, one would assume that in this case RIPK1-dependent apoptosis occurs instead of MLKL-dependent necroptosis; hence, confirmation of the involvement of necroptosis in luteolysis requires more in-depth experiments.

Recently, researchers described RIPK3-dependent cell death independent of MLKL activity in the murine CL [292]. They proposed a new model of RIPK3-dependent apoptosis which contributes to PGF2α-dependent luteolysis, but these data also imply that RIPK3 may switch to its canonical induction of necroptosis in some currently unknown conditions. Comparative proteomic data for several stages of CL development and regression identified necro(pto)sis among the most affected processes in the late luteal phase CL [293]. It is evident that the investigation of necroptosis in luteolysis is currently in its initial phase. Future studies should focus more on evaluating of definitive necroptosis features like MLKL activation and/or morphological changes.

## Pathologies associated with PCD deregulation in the ovary

Three waves of PCD in the normal ovary converge into an intricate cascade of events tightly controlled by hormones, cytokines, and local expression of cell death proteins. Therefore, it is not surprising that aberrations in PCD regulation result in severe impairments of ovarian tissue structure, the menstrual/estrous cycle, and reproductive function. Among numerous ovarian disorders, at least three are tightly associated with PCD deregulation: PCOS, premature ovarian insufficiency (POI), and malignant tumors. While some other pathologies like stromal hyperplasia, hyperthecosis, or pregnancy luteoma display tissue homeostasis disturbances and abnormal cell propagation, there are no evidences of them being driven by PCD aberrations [294].

### PCOS

PCOS is the most common endocrine/metabolic disorder diagnosed in women of reproductive age, affecting up to 20% of the population. It is a complex syndrome of unclear origin that manifests in hormonal disbalance (androgen and LH excess), obesity, diabetes, and infertility [295]. From the pathomorphological point of view, the most distinct PCOS feature is the accumulation of so-called “ovarian cysts”, which are in fact ovarian follicles at different stages of development [296]. The follicles in a polycystic ovary are unable to properly ovulate, and they do not undergo atresia, resulting in partial or complete abrogation of reproductive function.

The total amount of primary, secondary, and small antral follicles in a polycystic ovary is 2–6 times higher than in a normal one [297]. It seems logical to assume that such changes should be associated with the aberrations of either PGC depletion or antral follicle atresia. However, the events underlying PCOS development are much more controversial, and the molecular mechanisms remain elusive. Various PCOS animal models were developed; however, they often recapitulate only part of the PCOS phenotype, either in terms of morphologic ovarian changes or metabolic disturbances. The rodent models based on  the administration of aromatase inhibitor letrozole or constitutive LH overexpression seem to be the most adequate up to date [298]. Nevertheless, there is a clear lack of knowledge on PCOS-related changes during PGC depletion, and the available data mostly describe the changes observed during reproductive age.

Few reports have demonstrated that granulosa cells from polycystic ovaries have a lower apoptotic rate [299, 300] and increased expression of the pro-survival regulators BIRC3, EGFR, and HSP90B1 [299–301]. Moreover, expression of the mitogenic factor TGFα promotes the accumulation of antral follicles and a general PCOS-like state in mice [302]. In addition, polycystic ovaries display increased LHCGR expression, which may render them hypersensitive to LH effects (which may also be upregulated [303]), including ERK1/2- and AKT-mediated survival signals [255, 304, 305].

On the other hand, a growing number of studies have demonstrated that the accumulated follicles display an increased apoptotic rate. This activation of apoptosis can be associated with overexpression of established regulators of follicular atresia, including FasL [306], TNFα [303, 307], and FOXO3 [308]. Two other recurrent events observed in patients with PCOS and animal models of this disease are FSHR suppression [305, 309] and mitochondrial dysfunction [310, 311]. Deregulation of numerous other proteins (HMGB1, SH2B3, and USP25) and non-coding RNAs promotes apoptosis in polycystic ovaries [312–316], but their association with the process of normal follicular atresia is unclear. Such stimulation of apoptosis in PCOS follicles is without a doubt confusing, but it plays an important role in the development of anovulation and infertility by compromising oocyte maturation [114, 314].

Opposite changes in FSHR and LHCGR expression may underpin the distinct feature of PCOS—the inability of ovarian follicles to reach their outcome, either ovulation or atresia. FSH is required for the selection of dominant follicles, which subsequently induces atresia of smaller follicles [22], so a reduction in FSH sensitivity can potentially prevent this process and suspend the follicles in the early antral state. This idea was confirmed by a study of FSHR-antagonizing peptide that promotes the accumulation of secondary and pre-antral follicles in rat ovaries and a general PCOS-like state [317, 318]. Such follicles usually display increased apoptotic activity in preparation for atresia [114]; however, the increased LH sensitivity combined with LH excess may hinder mass cell death and, therefore, prevent atretic degradation.

Mitochondrial dysfunction in polycystic ovaries is a trending research area: researchers have linked mitochondrial dysfunction to apoptosis [311], as well as autophagy and ferroptosis. Active ROS generation and mitochondrial failure in PCOS can be associated with inefficient autophagy (and mitophagy in particular) [319, 320]; however, other recent reports describe increased autophagy/mitophagy [313, 321, 322]. Overexpression of several ferroptosis-associated genes was described in samples from patients with PCOS; unfortunately, their possible role in PCOS pathogenesis is not yet clear [323]. The discovery that ferroptosis is inhibited in granulosa cells from patients with PCOS via the circRHBG-miR-515-5p-SLC7A11 axis emphasizes the importance of PCD other than apoptosis in this disease and may provide new diagnostic molecular markers or therapeutic targets [214]. A recent report described an experimental attempt to treat PCOS in a mouse model by inducing ferroptosis [324]. The administration of n-3 PUFAs in the diet to dehydroepiandrosterone-treated mice activated Hippo signaling and YAP1 exocytosis, and suppressed NRF2, a protein that regulates critical ferroptosis-related genes (*GPX4*, *GSS*, *TFRC*, *HMOX1*, and others) [325], leading to the promotion of ferroptosis in ovarian granulosa cells. Considering the increasing interest toward autophagy and ferroptosis in the ovarian follicles, much can be expected from future studies in this direction. Current approaches for PCOS management are mostly focused on the compensation of hormonal disbalance and metabolic manifestations, while the accumulation of enlarged follicles is often considered a consequence that only impacts the fertility [326]. While the potential restoration of normal atresia in polycystic ovaries through selective PCD induction is not likely to improve fertility, it may contribute to the normalization of steroid hormone levels and therefore alleviate the severity of the disease symptoms.

### POI

The primordial follicles present at birth constitute the “ovarian reserve” that is supposed to be exhausted around the age of 45–50 years. If the follicles are depleted faster than usual, then this reserve can be completely exhausted at an earlier age. This condition is called POI and is diagnosed in 1–4% of women at an age of 40 years and 0.1% of women at an age of 30 years [327]. POI should be discerned from primary ovarian dysgenesis, which is caused by abnormal ovary development during embryogenesis and results in complete disruption of ovarian functions before birth [327]. POI should not be also confused with a poor ovarian response condition characterized by low sensitivity of the follicles to the hormonal signals and hindered ovulation [328]. True POI is associated with a decreased number of follicles at different stages of their development. This phenomenon can be caused by either impaired follicle formation or increased follicular degradation, with the latter being most relevant to the topic of this review.

Genome-wide studies have demonstrated a very prominent mutational heterogeneity of POI cases [329]; however, only some of the identified genes have been confirmed to be associated with PCD. The most well-established among them are *FIGLA*, *PGRMC1*, and *FMR1*. Folliculogenesis specific bHLH transcription factor (FIGLA) is an oocyte-specific transcription factor that regulates proper formation of the extracellular matrix around oocyte [330]. Mutations in the *FIGLA * gene do not clearly affect embryonic ovary development in mice but result in impaired formation of primordial follicles and drastic attrition of oocytes soon after the birth [331]. *FIGLA*  deficiency halts meiotic progression in PGCs and promotes DNA damage and subsequent apoptosis [332]. While complete *FIGLA* inactivation results in sterility [331], some mutations may exert milder effects and be associated with POI, as has been reported in Chinese and Indian populations [333, 334].

Progesterone receptor membrane component 1 (PGRMC1) is a non-canonical progesterone receptor that is expressed in granulosa cells and facilitates the anti-apoptotic and antimitotic effects of progesterone [335, 336]. Conditional *PGRMC1 * knockout in the murine reproductive tract results in subfertility without affecting the sensitivity to gonadotropin treatment [337]. Progesterone treatment can prevent granulosa cell apoptosis in response to oxidative stress, while PGRMC1 inhibition ablates this effect [338]. *PGRMC1*  expression is diminished in the granulosa cells of atretic follicles [339], suggesting that the inactivating mutations of this gene may promote atresia and thus contribute to POI development; however, this phenomenon has yet to be confirmed in large patient cohorts.

The *FMR1* (Fragile X messenger ribonucleoprotein 1) gene is located on the X chromosome and bears a repetitive stretch of 20–40 CGG triplets in its 5′ untranslated region. The number of these repeats can expand to 200+, result in high methylation and gene silencing, and cause mental retardation [340]. However, the carriers of a moderately increased number of CGG repeats (50–200, historically labeled as the “premutation” cohort) are prone to develop fragile X-associated POI [341]. It has no effect on the initial pool of primordial follicles [342, 343], but promotes their abnormally high recruitment and atresia rates during the pre-menopause period [341]. Mice carrying premutation *FMR1* develop POI and display increased apoptosis of granulosa cells [344]. Increased atresia of pre-antral and antral follicles results in low levels of anti-Müllerian hormone produced by granulosa cells; the deficiency of this hormone lifts the arrest of primordial follicle recruitment and quickly depletes the ovarian reserve [345].

Both *PGRMC1* and *FMR1* genes are located on the X chromosome. Besides the single mutations in these genes, there is a much more disruptive event: monosomy of the X chromosome also known as Turner syndrome [327]. It was initially thought to cause complete gonadal dysgenesis, but histologic studies of 45 XO fetuses have demonstrated that ovaries and PGCs develop normally during the first 3 months *in utero*, while primary follicle formation is impaired later and results in increased PGC attrition through apoptosis [346–348]. It is worth noting that the X chromosome also carries the anti-apoptotic *XIAP* gene, which is very important for preventing embryonic PGC depletion and postnatal antral follicle atresia [94, 165]. Hence, it is clear that the loss of one X chromosome in females disrupts multiple regulators of PCD in the ovary and almost inevitably results in POI [327].

Deregulation of autophagy can affect different stages of follicle development and, therefore, cause POI. Inactivating heterozygous mutations in the *ATG7* and *ATG9* genes are associated with a reduced number of primordial follicles [349], a finding consistent with experimental data obtained from *Atg7*-deficient mice [100, 101]. Inactivation of *Epg5* results in suppression of autophagy and promotion of granulosa cell apoptosis during the antral follicle stage, which may result in POI [196]. However, mutations in autophagy-related genes have not been reported among the most prevalent genetic aberrations in POI.

One more notable gene associated with POI is *BNC1*: researchers identified it as a potential POI determinant through genome-wide patient studies [350, 351] and functionally confirmed its role in a murine model [350]. Unlike the other genes mentioned above, *BNC1* deficiency triggers not apoptosis of granulosa cells, but ferroptosis of the oocyte itself, resulting in quick depletion of the ovarian reserve via follicular atresia [212].

Most comparative POI studies have focused on genome-based approaches and the identification of POI-associated mutations, while the data on gene expression patterns are scarce due to the nature of the disease: it is commonly diagnosed when the ovarian reserve is already depleted and there are no follicles left to investigate. However, several recent studies utilizing in vitro and in vivo models have allowed researchers to identify several other PCD-related genes and regulatory pathways potentially involved in POI development. Granulosa cells subjected to serum starvation in vitro display autophagy stimulation and reduced viability, and melatonin treatment rescues them by inhibiting autophagy through the PI3K-AKT-mTORC1 pathway (similarly to FSH effects; see “Regulation of PCD during follicular atresia - Autophagy” section) [352, 353]. Treatment of rats with 4-vinylcyclohexene diepoxide results in prominent miR-144 suppression, PI3K/AKT inhibition, autophagy induction, and POI development [354]. Taken together with the data on genetic inactivation of autophagic regulators presented above, autophagy should be tightly regulated to protect follicles from premature degeneration. These reports also suggest that treatment focused on stimulating the PI3K/AKT pathway (FSH and melatonin) could be used to prevent POI development. Because POI often involves ovarian tissue fibrosis, researchers developed a model of POI induction via cisplatin administration. Cisplatin promotes ferroptosis, and the investigators detected corresponding changes in the expression of ACSI4, ALOX15, SLC7A11, and GPX4 in the ovarian tissue alongside follicle development disorders and ovarian tissue fibrosis. The resulting POI and fertility reduction could be reverted by introducing ferroptosis-inhibiting vitamin E [355]. Considering the scarcity of available information on the role of apoptosis, ferroptosis and autophagy in POI, such discoveries are very important to understand the entire complex RCD system in the ovary. It also can be used as a foundation for the development of diagnostic and therapeutic approaches for POI.

### Tumors of ovarian origin

Malignant tumors represent the deadliest type of ovarian pathologies due to the lack of clear symptoms, concealed localization in the pelvic region, and the high risk of spreading to multiple adjacent organs. Like any cancer, the ovarian malignancies are tightly associated with various mechanisms of RCD suppression or evasion [356]. The entire field of RCD in ovarian cancer is enormous and lies far beyond the scope of this review. However, two specific cases should be mentioned because they are directly connected to normal PCD processes in the ovary.

Most ovarian tumors are high-grade serous carcinomas. A series of recent discoveries has demonstrated that these tumors commonly originate not from the ovary itself but rather from the fallopian tube epithelium. Therefore, they have no link to RCD events in the ovary [357]. Other ovarian cancer types are much rarer, and their cells of origin are usually unknown except for two very distinct subtypes: ovarian germ cell tumors (OGCTs) originating from PGCs and granulosa cell tumors (GCTs) [358]. Due to the very limited availability of samples, information on mechanisms underlying the development of these tumors is scarce.

OGCTs are usually diagnosed in the juvenile age and include several subtypes: dysgerminomas, immature teratomas, yolk sac tumors, and embryonal carcinomas [358]. While these malignancies differ significantly in their structure and prognosis, they share a common trait of frequent chromosome 12 amplification or isochromosome formation [359–361]. Chromosome 12 carries such relevant genes as *KITLG* and *KRAS*, which are crucial for PGC survival in the embryonic ovary [60, 61]. Thus, it is not surprising that their amplification promotes abnormal PGC proliferation. Moreover, OGCTs often display amplification or activating mutations of the KIT receptor and KRAS, thus further promoting PGC survival [359, 362, 363]. The tumor-driving roles of KIT, KITLG, and KRAS remain to be confirmed in animal models, but the well-established teratogenic effect of BCL-2 specifically expressed in granulosa cells [158] clearly illustrates that inhibition of apoptosis in PGCs promotes their malignisation. Unfortunately, due to the rarity of OGCTs and limited interest to their recapitulation in the laboratory animals, this intriguing model is not widely used.

GCTs arise either around menopause (45–50 years) or, in rare cases, the juvenile period (before 20 years). These two forms are histologically distinct, but they both display a common C134W mutation in the FOXL2 protein [364, 365]. Surprisingly, this mutation does not seem to have any significant impact on FOXL2 functions [366]. However, a transcriptomic study demonstrated that clinical GCT samples display changes in FOXL2 targets associated with reduced cell death and increased proliferation [367]. Wild-type FOXL2 exerts pro-apoptotic effects upon granulosa cells through caspase-8, BID, and BAK, and is associated with increased levels of TRAIL and CD95/Fas/Apo-1 expression. These associations are lost in FOXL2^C134W^ granulosa cells [368]. FOXL2 activity is crucial for granulosa cell differentiation and secondary follicle formation, but its exact role in follicle survival/atresia remains elusive [369]. Despite the lack of mutations tightly associated with GCT cases in patients, several animal models were developed; in many of them, the promotion of tumor development is facilitated by inactivation of well-known mediators of follicular atresia (see “Regulation of PCD during follicular atresia - Apoptosis” section), such as α-inhibin, SMAD1/5, or FOXO1/3 [370]. These models additionally highlight the importance of PCD in the maintenance of normal ovarian function and hint at potential targeting of antral follicle-specific PCD regulation pathways for GCT treatment.

## Conclusion

The regulation of PCD in the normal ovary is an extremely complicated process, in which the successful completion of each step is essential for the onset of the next development stage. Without proper PGC attrition, it is impossible to form stable primordial follicles; antral follicle atresia is a part of dominant follicle selection and subsequent ovulation; and CL degradation concludes each ovarian cycle and resets it. The disruption of this order of events can be devastating to the patient’s reproductive function and general health.

Over the last 50 years, the anatomical, physiological, and molecular events underlying PCD in the ovary have been established in great detail, and the new aspects related to less common RCD modes like necroptosis and ferroptosis are being reported as our knowledge expands. Table 1 provides a structured summary of numerous signaling pathways involved in PCR-dependent processes in the mammalian ovary and clearly illustrates their overwhelming complexity and interconnections. However, the available information on the possible pathologic consequences of PCD deregulation in the ovary is still incomplete and fragmentary. Most studies focused on PCOS, POI, or rare ovarian tumors are devoted to finding new diagnostic or therapeutic approaches regardless of the initial disease causes or mechanisms involved in their development. While the bias may be explained by the tremendous heterogeneity of the genetic landscape of these disorders, the diverse stimuli still converge to very distinct pathophysiological manifestations that are clearly connected to PCD impairments. It is notable that almost all studies exploring PCD aspects of ovarian disorders were published over the last decade, indicating the evident shift in the research interest. We expect that the forthcoming advances in this area may represent a new phase in managing ovarian pathologies, with a solid emphasis on PCD-associated traits instead of a symptomatic approach.

**Table 1:** Major signaling pathways controlling different PCD-related events in the mammalian ovary

Pathway	Ovarian event	PCD type	Upstream regulators	Downstream effectors	Effect on ovarian cells	References
PI3K/AKT	PGC depletion	Apoptosis	KITLG	Upregulation of BCL-2, BCL-XL, MCL-1 Inactivation of BIM, BAX, BAD, and FOXO3	Pro-survival	[72, 77–80]
	Follicular atresia	Apoptosis	FSH, IGF, EGF, bFGF	Upregulation of NFκB, cFLIP_L_, BCL-2, MDM2, and XIAP Inactivation of caspase-8, BIM, BAX, BAD, FOXO1, and FOXO3	Pro-survival	[77, 141–144, 150, 157–170, 173–177]
		Autophagy	FSH	Downregulation of MAP1LC3-II, BECN1, ATG7, and ATG12	Pro-survival	[188, 191]
	Luteolysis	Apoptosis	PGF2α	Inhibition of AKT Upregulation of BAX	Pro-apoptotic	[247]
PLC/PKC	Follicular atresia	Apoptosis	FSH	Stabilization of cFLIP_S_	Pro-survival	[143]
	Luteolysis	Apoptosis	PGF2α	Activation of NFAT	Pro-apoptotic	[231, 242, 243]
Ca^2+^/AMPK	Luteolysis	Autophagy	PGF2α	Activation of BECN1 Upregulation of MAP1LC3-II	Pro-autophagic	[286]
MEK/ERK	PGC depletion	Apoptosis	KITLG	Activation of RPS6KA1, MYC, STAT1/3	Pro-survival	[72, 77]
	Follicular atresia	Apoptosis	FSH through β-arrestin	Activation of RPS6KA1, MYC, STAT1/3	Pro-survival	[150, 152]
	Luteolysis	Apoptosis	LH/hCG through β-arrestin	Activation of RPS6KA1, MYC	Pro-survival	[255, 261]
		Autophagy	PGF2α	Upregulation of MAP1LC3-II	Pro-autophagic	[288]
cAMP/PKA	Follicular atresia	Apoptosis	FSH	Phosphorylation of CREB, ERK1/2, AKT, GSK3B, β-catenin, FOXO1, p38 MAPK Downregulation of BIM	Pro-survival	[153–155]
	Luteolysis	Apoptosis	LH/hCG	Progesterone synthesis stimulation Activation of CREB, GSK3B, and β-catenin Upregulation of LHCGR, BCL-2, MCL-1, survivin, STAR, and PGE2α Downregulation of BAX and CD95/Fas	Pro-survival	[156, 256–258, 262, 263, 265–269, 277]
			PGE2α	Upregulation of LHCGR, BCL-2, STAR	Pro-survival	[273, 277]
		Autophagy	LH/hCG	Downregulation of MAP1LC3-II	Pro-survival	[286]
Estrogen signaling	Luteolysis	Apoptosis	Progesterone	Downregulation of CD95/Fas Upregulation of BCL-2	Pro-survival	[270–273]
HIF1α	Follicular atresia	Autophagy	Hypoxic stress, FSH	Upregulation of PINK1, Parkin, and BNIP3	Pro-survival	[197–201]
	Luteolysis	Autophagy	PGF2α	Upregulation of BNIP3	Pro-autophagic	[287]
JAK/STAT	PGC depletion	Apoptosis	LIF	Upregulation of BCL-2, BCL-XL, MCL-1	Pro-survival	[74–76]
	Follicular atresia	Apoptosis	IFNγ	Upregulation of CD95/Fas	Pro-apoptotic	[125–128]
	Luteolysis	Apoptosis	PGF2α	Activation of STAT3 Upregulation of SOCS3	Not defined/pro-apoptotic	[247]
			IFNγ	Upregulation of CD95/Fas	Pro-apoptotic	[224, 232]
CHK2/p53/p63	PGC depletion	Apoptosis	Lack of growth factors, nutrient starvation, stress	Activation of p53/TAp63 Upregulation of NOXA, BBC3, and BAX	Pro-apoptotic	[35, 63–65, 84, 85]
Death receptor/FADD/caspase-8	PGC depletion	Apoptosis	TNFα	Activation of BAK, BAX, and caspase-3	Pro-apoptotic	[87]
			TRAIL	Activation of BAK, BAX, and caspase-3	Pro-apoptotic	[88]
	Follicular atresia	Apoptosis	FasL	Activation of BAK, BAX, and caspase-3	Pro-apoptotic	[117–126]
			TNFα	Activation of BAK, BAX, and caspase-3 Downregulation of BCL-2	Pro-apoptotic	[126, 131–133]
			TRAIL/DR4/5 receptors	Activation of BAK, BAX, and caspase-3	Pro-apoptotic	[88, 134–136]
	Luteolysis	Apoptosis	FasL	Activation of BAK, BAX, and caspase-3	Pro-apoptotic	[123, 125, 228, 229]
			TNFα	Activation of BAK, BAX, and caspase-3 Upregulation of CD95/Fas	Pro-apoptotic	[224, 226, 229, 231, 232]
NFκB-RelA	Follicular atresia	Autophagy	FSH	Activation of JNK Upregulation of MAP1LC3-II	Pro-autophagic, pro-survival	[193]
	Luteolysis	Apoptosis	IFNγ	Upregulation of CD95/Fas	Pro-apoptotic	[224, 232, 235, 236]
			TNFα	Upregulation of CD95/Fas	Pro-apoptotic	[224, 232, 235, 236]
RIPK1/RIPK3	Follicular atresia	Apoptosis	SIRT1	Activation of caspase-3	Pro-apoptotic	[210]
		Necroptosis	AChE-R-derived peptide	Activation of MLKL	Pro-necroptotic	[207–209]
	Luteolysis	Apoptosis	PGF2α	Activation of RIPK3 and caspase-8	Pro-apoptotic	[292]
		Necroptosis	AChE-R-derived peptide	Activation of MLKL	Pro-necroptotic	[207]
			Ceramide	Activation of MLKL	Pro-necroptotic	[289]
Iron turnover	PGC depletion	Ferroptosis	Transferrin, PCBP2	SLC7A11	Not defined	[110, 111]
NF2/YAP	Follicular atresia	Ferroptosis	BNC1 inactivation	Downregulation of GPX4 Upregulation of NOX1, COX2, TFRC and ACSL4	Pro-ferroptotic	[212]
x_c_^−^ system	Follicular atresia	Ferroptosis	circRHBG-miR-515-5p	Cystine metabolism	Pro-ferroptotic	[214]
Pathways are not determined up to date	PGC depletion	Apoptosis	Starvation at birth	Upregulation of BAX	Pro-apoptotic	[103]
		Autophagy	Starvation at birth	Upregulation of MAP1LC3-II and LAMP1	Pro-autophagic, contradictory effects	[103, 105]
	Follicular atresia	Apoptosis	Oxidative stress	Upregulation of BIM and caspase-3 Activation of caspase-3	Pro-apoptotic	[195]
		Autophagy	Oxidative stress	Activation of JNK Upregulation of BECN1, ATG5, ATG7, and MAP1LC3-II	Pro-autophagic, pro-ADCD	[194, 195]
	Luteolysis	Apoptosis	PGF2α	Upregulation of FasL, CD95/Fas, BAX, IFNγ, and TNFα Activation of caspase-3	Pro-apoptotic	[221, 244, 245, 248]
			TNFα	Stimulation of PGF2α secretion	Pro-apoptotic	[246]
		Autophagy	PGF2α	Upregulation of MAP1LC3-II	Pro-autophagic, pro-survival	[248]

## A comment from the authors

We sincerely regret that, due to the reasonable limitations of manuscript length and reference number, we could not properly cite many of the original research papers describing the facts and events discussed above. The field of PCD in the mammalian ovary is immensely vast and accumulated an overwhelming amount of information over more than half of the century. We therefore attempted to highlight the most ovary-relevant and up-to-date reports, while referring to the review articles in discussions of more general aspects.

## Data Availability

No unique dataset was generated during preparation of this manuscript.

## Abbreviations

RCD: Regulated cell death
PCD: Programmed Cell Death
PGCs: Primordial germ cells
FSH: Follicle-stimulating hormone
LH: Luteinizing hormone
CL: Corpus luteum
PGF2α: Prostaglandin F2α
CG/hCG: Chorionic gonadotropin/human chorionic gonadotropin
BCL-2: B cell leukemia/lymphoma 2
BIM (BCL2L11): BCL2 interacting mediator (BCL2-like 11)
NOXA (PMAIP1): Phorbol-12-myristate-13-acetate-induced protein 1
BBC3 (PUMA): BCL2 binding component 3 (p53 upregulated modulator of apoptosis)
BAD: BCL2 associated agonist of cell death
BCL-XL (BCL2L1): B-cell lymphoma extra large (BCL2-like 1)
BCL-2A1: BCL2 related protein A1
MCL-1: Induced myeloid leukemia cell differentiation protein
BAX: BCL2 associated X; BCL2-like protein 4
BAK: BCL2 homologous antagonist/killer
APAF1: Apoptotic peptidase activating factor 1
TNF: Tumor necrosis factor
CD95/Fas/Apo-1: Tumor Necrosis Factor Receptor Superfamily Member 6/ Fas cell surface death receptor
TNFR1/2 (TNFRSF1A/1B): TNF receptor superfamily member 1 A/1B
TRAIL (TNFSF10): TNF superfamily member 10
FADD: Fas associated via death domain
TRADD: TNFRSF1A associated via death domain
BID: BH3 interacting domain death agonist
ADCD: Autophagy-dependent cell death
PI3K/PI3KC3: Phosphatidylinositol 3-kinase
AKT: Serine/threoninespecific protein kinase 1
mTOR: Mammalian target of rapamycin
AMPK (PRKAA1): Protein kinase AMP-activated catalytic subunit alpha 1
ULK: Unc-51 like autophagy activating kinase
BECN1: Beclin 1
ER: Endoplasmic reticulum
ATG3/4/5/7/9/12/16: Autophagy related 3/4/5/7/9/12/16
MAP1LC3: Microtubule associated protein 1 light chain 3 (also known as LC3)
LC3-II (MAP1LC3A): Microtubule Associated Protein 1 Light Chain 3 Alpha
LAMP1/2: Lysosomal associated membrane protein 1
RIPK1/3: Receptor interacting serine/threonine kinase 1/3
MLKL: Mixed lineage kinase domain like pseudokinase
FasL: Fas ligand
TRAF2: TNF receptor associated factor 2
cIAP1/2 (BIRC2/3): Baculoviral IAP repeat containing 2/3
IAP: Inhibitor of apoptosis protein
NFkB: Nuclear factor kappa B
ROS: Reactive oxygen species
PUFAs: Polyunsaturated fatty acids
MUFAs: Monounsaturated fatty acids
GSH: Glutathione
GPX4: Glutathione peroxidase 4
RSL3: RAS-selective lethal
KITLG (SCF): KIT ligand
LIF: Leukemia inhibitory factor
IGF1: Insulin like growth factor 1
cAMP: Cyclic adenosine monophosphate
MEK: ERK kinase
ERK (MAPK1): Extracellular signal-regulated kinase (mitogen-activated protein kinase 1)
c-KIT (KIT/CD117): KIT proto-oncogene, receptor tyrosine kinase
JAK: Janus kinase
STAT3: Signal transducer and activator of transcription 3
FOXO1/3: Forkhead box O1/3
CHK2: Checkpoint kinase 2
GSK3B: Glycogen synthase kinase 3 beta
XIAP: X-linked inhibitor of apoptosis
PDK1: Pyruvate dehydrogenase kinase 1
TSC1: TSC complex subunit 1
SIRT1: Sirtuin 1
ATPase: Adenosine 5'-triphosphate
IFNγ: Interferon-γ
DR4/5: Death receptor 4/5
DcR1/2: Decoy receptor 1/2
DISC: Death-inducing signaling complex
cFLIP (CFLAR): CASP8 and FADD like apoptosis regulator
PKC: Protein kinase C
FSHR: FSH receptor
PKA: Protein kinase A, cAMP-dependent
MDM2: Mouse double minute 2 homolog
TGFα/β1: Transforming growth factor alpha/beta 1
EGF: Epidermal growth factor
FGF: Fibroblast growth factor
JNK (MAPK8): Mitogen-activated protein kinase 8
HIF1α: Hypoxia inducible factor 1 subunit alpha
PINK1: Phosphatase and tensin homolog induced kinase 1
BNIP3: BCL2 interacting protein 3
VEGFA: Vascular endothelial growth factor A
AChE-R: Acetylcholinesterase
PARP: Poly (ADP-ribose) polymerase
BNC1: Basonuclin zinc finger protein 1
NF2: Neurofibromin 2
YAP: Yes associated protein
SLC7A11: Solute carrier family 7 member 11
PCOS: Polycystic ovary syndrome
SAMP: Senescence-accelerated mouse strain
PTGFR: PGF2α receptor
PLC: Phospholipase C
PGE2: Prostaglandin E2
MAPK: Mitogen-activated protein kinase
LHCGR: LH/CG receptor
STAR: Steroidogenic Acute Regulatory Protein
PTGER: PGE2 receptors
MAPK: Mitogen-Activated Protein Kinase
POI: Premature ovarian insufficiency
BIRC3: Baculoviral IAP repeat containing 3
EGFR: EGF receptor
HSP90B1: Heat shock protein 90 beta family member 1
HMGB1: High mobility group box 1
SH2B3: SH2B adaptor protein 3
USP25: Ubiquitin specific peptidase 25
NRF2 (NFE2L2): Nuclear factor, erythroid derived 2, like 2
FIGLA: Folliculogenesis specific bHLH transcription factor
PGRMC1: Progesterone receptor membrane component 1
FMR1: Fragile X messenger ribonucleoprotein 1
OGCTs: Ovarian germ cell tumors
GCTs: Granulosa cell tumors
